# Catalytic Pyrolysis of Polypropylene to Benzene, Toluene, and Xylene (BTX) Using a Double-Fluidized-Bed Reactor

**DOI:** 10.1021/acs.energyfuels.4c05316

**Published:** 2025-02-07

**Authors:** Hongqi Wang, Matthijs van Akker, Jozef G. M. Winkelman, André Heeres, Hero Jan Heeres

**Affiliations:** † Green Chemical Reaction Engineering, Engineering and Technology Institute Groningen, University of Groningen, Nijenborgh 4, 9747 AG Groningen, The Netherlands; ‡ BioBTX BV, Zernikelaan 17, 9747 AA Groningen, The Netherlands; § Hanze University of Applied Sciences, Zernikeplein 11, 9747 AS Groningen, The Netherlands

## Abstract

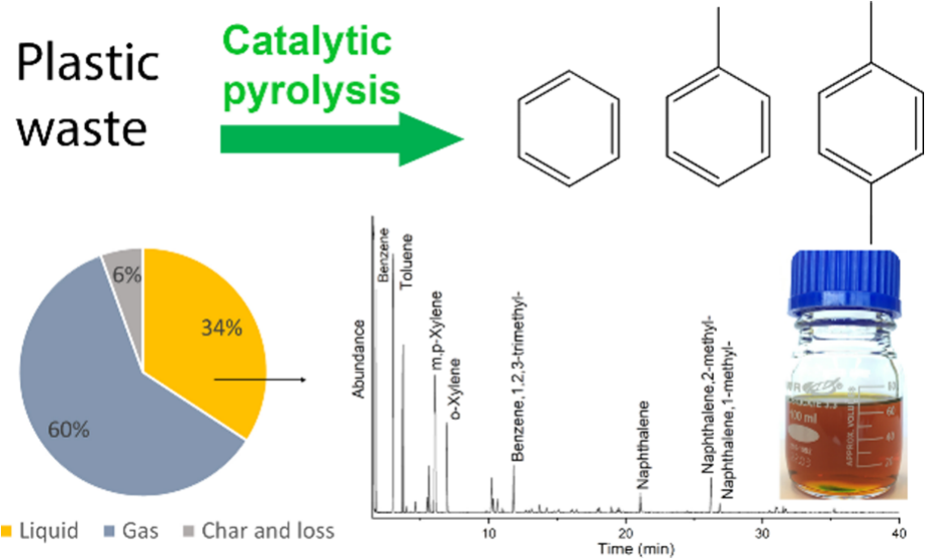

The current plastic value chain is highly linear, leading to large amounts of waste plastics that harm the environment and human health. Recycling is required, and among the options, catalytic pyrolysis is particularly suited to convert polyolefin-rich plastic waste into useful chemicals such as benzene, toluene, and xylene (BTX). In this paper, we demonstrate *ex situ* catalytic pyrolysis of polypropylene in a continuous double-fluidized-bed reactor to produce BTX. The optimal pyrolysis temperature in the first fluidized-bed reactor was 550 °C, giving a BTX yield of 22.3 wt % (based on PP input). Lowering the nitrogen flow rate and the use of smaller catalyst particle sizes favor BTX formation. Our novel reactor concept showed good operational stability at longer times on stream (TOS, 10 h). Catalyst activity was slightly reduced during TOS, as is evident from a small decrease in BTX yields. Detailed catalyst characterization studies showed that coke formation is the main reason for catalyst deactivation. In addition, chemoselectivity was also a function of TOS and the selectivity to benzene and toluene decreased, while higher amounts of xylenes were formed.

## 1 Introduction

Plastic is widely used due to its unique properties such as low weight, low cost, durability, and versatility.^1^ The ever-increasing global plastic consumption combined with ineffective waste management policies have led to the formation of enormous amounts of plastic waste that mainly ends up in landfills or rivers and oceans.^2^ The extremely slow degradation rate of plastic waste in the natural environment^3^ and the current situation of low recycling may threaten the environment and even human health through the food chain at various scales, such as microplastics (<5 mm)^2^ or nanoplastics (<6 nm).^4^ Therefore, it is essential to prevent disposing of plastic waste in landfills or oceans by converting it into base chemicals through efficient recycling strategies.^5^

Catalytic pyrolysis is a very promising and effective technique to upcycle polyolefin (PO)-rich plastic waste to produce valuable chemicals such as benzene, toluene, and xylene (BTX).^6,7^ Generally, the pyrolysis vapor derived from the thermal decomposition of PO-rich plastic waste can be converted into BTX using catalysts in an *in situ* or *ex situ* mode.^8^ Zeolite catalysts, especially the H-ZSM-5 family, are generally employed to produce BTX due to their high aromatization performance, resulting from high acidity and microrange-sized pores.^9,10^

Reactor configuration has a major effect on product yields during pyrolysis processes.^11^ In general, heat input is a major issue, particularly for scale-up.^12^ In addition, mass transfer limitations may play a role, especially for *in situ* catalytic pyrolysis in which plastic solid/melt/vapor is in direct contact with the solid catalyst.^13^ Another issue is pressure build-up/blockage, particularly encountered when using fixed-bed reactor configurations due to coke and char formation.^14^ To obtain high product yields for PO-rich plastic waste, reactor configurations with high heat and mass transfer are required to overcome these challenges.^15^

We propose a new reactor concept for the *ex situ* catalytic pyrolysis of waste PO-rich plastics involving two fluidized beds in series. In the first reactor, the POs are thermally pyrolyzed in a fluidized bed with sand particles as the heat transfer medium. The vapors formed are passed to a second reactor containing the aromatization catalyst. In this configuration, the advantages of common fluidized-bed reactors such as good heat and mass transfer are used in both the pyrolysis and aromatization reactors.^16^ The other advantages of using double-fluidized-bed reactors include independent temperature control and process integration. For example, when a three-stage fluidized-bed reactor was used for the aromatization of *n*-pentane, enhanced yield was achieved by application of different temperatures in the three catalyst zones.^17^ Another example involves the chemical recycling of poly(methyl methacrylate). In this case, the polymer was first depolymerized into methyl methacrylate in a fluidized-bed reactor and subsequently hydrolyzed to methacrylic acid with high yield in a fixed-bed reactor operated in tandem.^18^

We present a proof of concept demonstrating the use of a double-fluidized-bed reactor system, arranged in series, to convert PO-rich waste plastics into value-added BTX on a laboratory scale. Polypropylene (PP) was used as a typical model plastic because it is a major component in household plastic waste.^4,19^ The optimal operating conditions for high BTX yield were investigated by exploring the effects of the pyrolysis temperature, flow rate of the fluidization gas, and catalyst particle size on BTX yields. Operational and catalyst stability was examined by performing experiments at extended times on stream (TOS) of up to 10 h. To gain insight into possible deactivation pathways, after reaction, the catalyst was characterized in detail.

## 2 Experimental Section

### 2.1 Materials

Granular PP pellets were provided by Hellenic Petroleum and used as received (average pellet diameter, 3.6 mm). Sand for the pyrolysis reactor was supplied by Sigrano Nederland B.V. A sieve fraction of 45–100 μm was used. H-ZSM-5 (SiO_2_/Al_2_O_3_ = 38) with 30 wt % pseudoboehmite as the binder was purchased from ACS material. The original pelletized catalyst was crushed and sieved, and a fraction of 45–100 μm was used for the catalytic experiments. Before use, the sieve fraction was calcined in air at 550 °C for 8 h. *n*-Octane (>97%) was bought from Sigma-Aldrich and used without further treatment. THF (>99.85%) was purchased from Boom B.V. Nitrogen gas (5.0) was provided by SOL Nederland B.V.

### 2.2 Pyrolysis Setup

**Figure 1:**
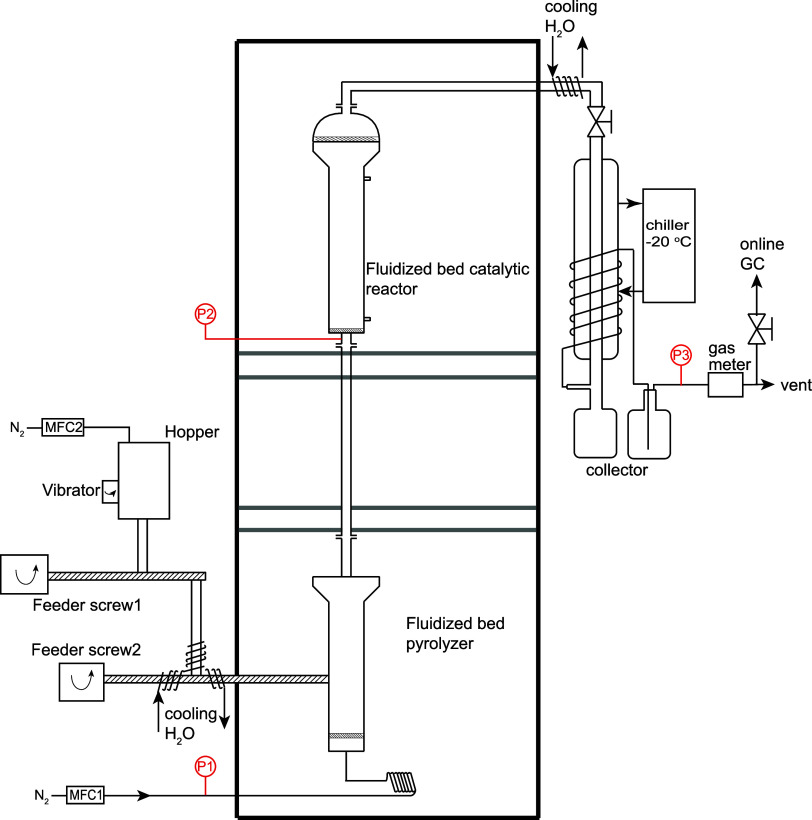
Double-fluidized-bed reactor configuration used for the *ex situ* catalytic pyrolysis of PP.

Noncatalytic/catalytic pyrolysis experiments of PP were conducted in a double-fluidized-bed reactor, as shown in Figure 1. This reactor configuration ensures high heat and mass transfer efficiencies due to the intense mixing of sand/catalyst of small particle sizes with the reactant plastic or pyrolysis vapor. PP particles were fed into the pyrolysis reactor through two screws under a N_2_ atmosphere. The first screw is used to control the feed rate, whereas the second is operated at full speed to avoid blockages. The latter is also cooled to avoid both melting of the plastic and formation of a viscous paste that would hamper feeding. N_2_ was used as the fluidization gas and preheated to the preset temperature before entering the first fluidized-bed reactor. A porous stainless steel plate distributor (pore size of 40 μm) was used as the bottom plate. The first fluidized-bed reactor contains sand (100 g, 45–100 μm sieve fraction). The second catalytic aromatization reactor contains the zeolite catalyst (100 g, 45–100 μm sieve fraction). The product vapor is sequentially condensed in two stages, one cooler with cold water at 25 °C and a second at −20 °C. The liquid product from the two collection flasks was combined for mass balance calculations and further analysis. The amount of noncondensable gases was measured by a flowmeter and analyzed online using a GC-FID/TCD to determine its composition. The experiments were conducted in duplicate. The standard deviations were calculated and are shown as error bars in the relevant figures.

### 2.3 Product Analyses

The gas-phase product was analyzed by an online GC-FID/TCD (Thermo Scientific Trace 1300) that is composed of two channels. A Restek packed column (HS-Q 60–80, 0.25 m, 1.0 mm ID, 1/16 in. OD) is installed as a precolumn. The exit stream is then split and fed to a Rt-Qbond (30 m × 0.53 mm ID × 20 μm) followed by a Rt-MSieve (30 m × 0.53 mm ID × 50 μm) for TCD detection and a second channel with an Rtx-1 (30 m × 0.53 mm ID × 3 μm) column for FID detection. The oven temperature program started with an isothermal stage of 35 °C for 1.5 min. Then, the oven temperature was increased to 100 °C at 25 °C/min and kept at this temperature for 7 min, followed by an increase to 165 °C at a rate of 30 °C/min.

The BTX components and other aromatics in the liquid product were quantified by a GC-FID instrument (Agilent Technologies 7890B) equipped with an Agilent Technologies DB-5 (15 m × 0.32 mm × 0.25 μm) column. Before injection, the liquid product was diluted using THF with octane as the internal standard. Quantification of the amounts of BTX was performed using an internal standard method with *n*-octane. A 5-point calibration curve was used to determine the relative response factors. The diluted solution was injected at 280 °C with a split ratio of 1:50 and a helium flow rate of 1 mL/min. The oven was heated from 40 to 250 °C at a rate of 5 °C/min and then kept at 250 °C for 3 min.

The composition of liquid products was analyzed using GC-MS (HP 6890 GC and HP 5973 MSD) equipped with a Restek Rxi-5Sil MS column (30 m × 0.25 mm × 0.25 μm). A THF-diluted sample (1 μL) was injected at 280 °C with a split ratio of 1:50. The oven temperature was maintained at 40 °C for 5 min and then heated to 250 °C at a rate of 3 °C/min.

A TGA (TGA 4000 from PerkinElmer) was used to determine the coke content of the spent catalyst. The sample was heated to 900 °C at 10 °C/min in a flow of 50 mL/min of air. The coke content was defined as the weight percentage difference between 100 and 900 °C to exclude the effects of water. Before TGA analyses, the catalyst samples were stored in an oven at 105 °C for extended periods to reduce free and bound water levels as much as possible. TGA was also used to analyze the pyrolysis behavior of the PP feed and to determine the distillation curve of the liquid products. For these purposes, the samples were heated to 900 °C at a rate of 10 °C/min in a flow of 50 mL/min of N_2_.

A NH_3_-TPD (AutoChem II 2920 from Micromeritic) was used to quantify the total acidity of the catalyst samples. The sample was heated to 550 °C at a rate of 10 °C/min in a flow of 50 mL/min of He and maintained under these conditions for 1 h. Subsequently, the sample was cooled to 100 °C. 10% NH_3_ in He of 50 mL/min was introduced, and the sample was maintained under these conditions for 1 h. The ammonia was replaced with He (50 mL/min) for 1 h to remove weakly adsorbed NH_3_. The catalyst was then heated to 550 °C at a rate of 10 °C/min and maintained at this temperature for 10 min. The desorbed products were quantified by using a TCD detector calibrated for NH_3_.

Nitrogen adsorption–desorption isotherms were obtained using an ASAP 2420 Surface Area and Porosity Analyzer (Micromeritics) at 77 K. The catalyst was heated to 450 °C and maintained under these conditions for 4 h to degas. The surface area was acquired using the Brunauer–Emmett–Teller (BET) method, and the pore size distribution was determined by the Barrett–Joyner–Halenda (BJH) method.

### 2.4 Definitions

The yield of products such as gas, liquid, and BTX is defined as
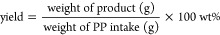
1

The selectivity to individual BTX compounds is defined as
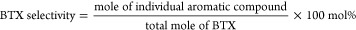
2

## 3 Results and Discussion

### 3.1 Characterization of the PP Feed

Relevant properties of the PP feed used in this study were determined by elemental analyses and TGA. The elemental composition results are depicted in Table 1 and, as expected, show that the main elements are carbon and hydrogen. Minor amounts of N and S were present (<0.2 wt %), most likely from additives in the PP feed. The ash content is 0.08 wt % as determined by TGA (900 °C, air).

**Table 1:** Ultimate Analysis of the PP Feed

element	C	H	N	S	ash content
content (wt %)	85.28 ± 0.01%	13.49 ± 0.14%	0.11 ± 0.01%	0.20 ± 0.04%	0.08%

**Figure 2:**
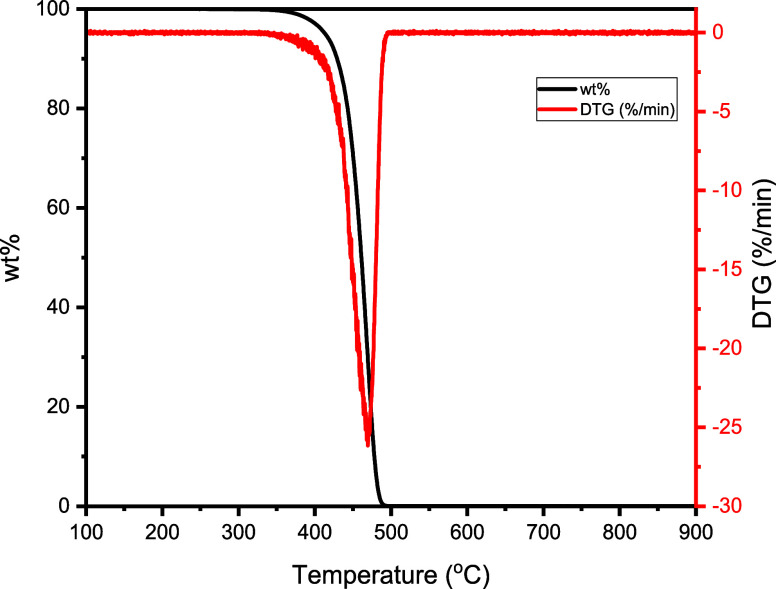
TGA and DTG curves of PP pyrolysis at 10 °C/min in N_2_.

A TGA under N_2_ was also conducted to determine the pyrolysis behavior of PP used in this study. As shown in Figure 2, PP was quantitatively pyrolyzed at 500 °C (10 °C/min heating rate). A maximum degradation rate of 26 wt %/min was obtained at 470 °C. As such, 500 °C was selected as the minimum pyrolysis temperature for the experiments in this study to ensure quantitative decomposition of PP in the pyrolysis reactor.

### 3.2 Benchmark Experiment

**Figure 3:**
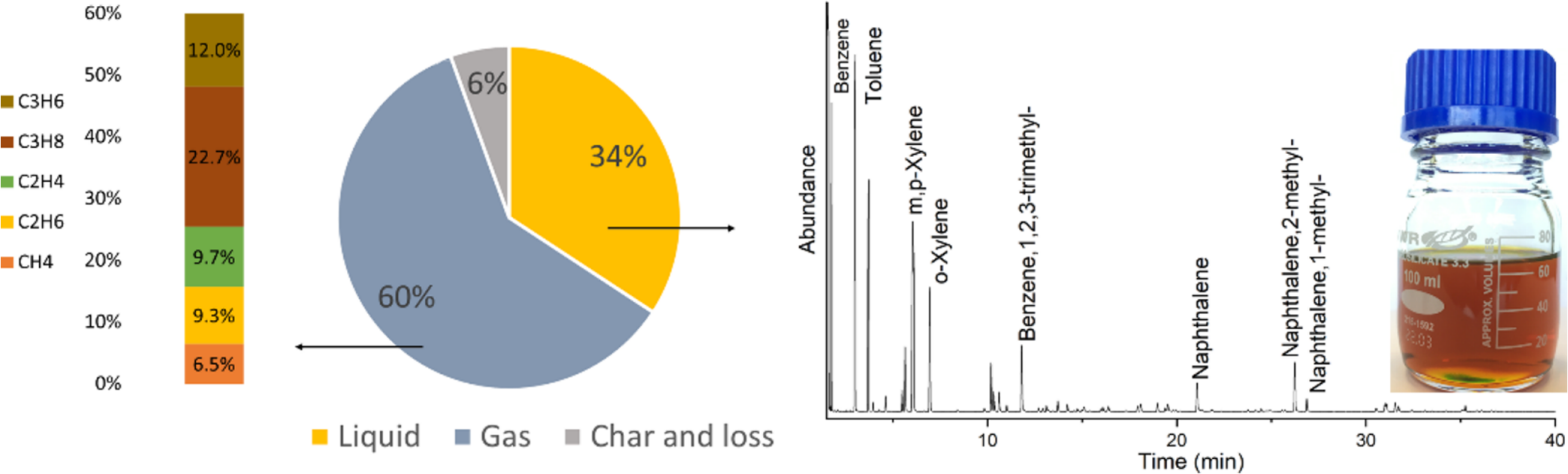
Mass balance and product composition of the benchmark experiment (Table 2).

The first double-fluidized-bed reactor experiments were carried out at equal temperatures for the pyrolysis and aromatization reactors (550 °C, benchmark conditions). Other relevant conditions are given in Table 2. The pyrolysis temperature is based on previous studies in our group.^20^ The temperature of the aromatization reactor was fixed during this investigation to reduce the number of variables. A value of 550 °C was selected as this is a common value in catalytic pyrolysis studies using H-ZSM-5 based catalysts.^21−24^ The nitrogen flow rate is based on cold-flow studies and theoretical calculations to ensure fluidization behavior in both reactors (Section S1). The reactor was run for a TOS of 2 h without any operational issues. After the reaction, the gas and liquid phases products were quantified and used for mass balance calculations. The liquid yield was 34 wt %, whereas substantially more gas was formed (60 wt %; see Figure 3). The observed gas yield aligns closely with the reported yield of 56% for PP pyrolysis catalyzed by H-ZSM-5.^25^ Solids could not be quantified accurately, though they are less than 6 wt % based on mass balance considerations.

**Table 2:** Experimental Process Conditions

conditions	benchmark	ranges
pyrolysis reactor temperature (°C)	550	500–700
aromatization reactor (°C)	550	550
nitrogen flow rate (NL/min)	0.66	0.66, 1.32, 1.98
catalyst particle size (μm)	45–100	45–100, 75–150
PP feed rate (g/h)	150	150
time on stream (h)	2	up to 10
catalyst intake (g)	100	100
sand intake (g)	100	100

The light yellow, transparent liquid-phase product was analyzed in detail using various techniques (*vide infra*). The BTX yield was 22.3 wt % (on PP intake, GC; see Figure 3 for details), the main component being toluene. In addition to BTX, significant amounts of other aromatics such as naphthalenes were also present in the liquid (Figure 3).

The liquid and BTX yields for representative (catalytic) pyrolysis studies on waste plastics are summarized in Table 3 for comparison. Proper comparison is hampered by the differences in equipment sizes, experimental conditions, and feed composition. The BTX yield is comparable to values reported by Anunziata et al.^26^ using a heated flow reactor filled with HDPE and a H-ZSM-11 catalyst (24.7 wt %; see Table 3).

**Table 3:** Representative (Catalytic) Pyrolysis Experiments of Waste Plastic Using ZSM-Type Catalysts

reactor configuration	reactor scale	relevant conditions	liquid yield (%)	BTX yield (%)	BTX selectivity	ref
batch, fixed-bed reactor	2 g of electronic plastic waste	500 °C, 200 mL/min N_2_, 4 g of H-ZSM-5	50	N/A	15 (FID area%)	(27)
batch, fluidized-bed reactor	PP	390 °C, 600 mL/min N_2_, PP to H-ZSM-5 catalyst ratio = 30 wt %, total time of collection = 30 min	38.8	1.8	N/A	(25)
single-pass flow reactor, batch	HDPE	500 °C, H-ZSM-11 catalyst	44.8	24.7	N/A	(26)
batch, fixed-bed reactor	LDPE	600 °C, 10 g of ZSM-5	75	TX: 0.88	N/A	(28)
batch, fluidized-bed reactor	PP	360 °C, 570 mL/min N_2_, PP to catalyst = 40 wt %	2.31	1.82	N/A	(29)
horizontal tubular reactor	HDPE, LDPE	2 kg/h, H-ZSM-5 catalyst = 4 wt % on feed	61.4	31.78 of oil	N/A	(30)

The gas phase consists mainly of propane, followed by propene, ethene, ethane, and methane, due to the excessive cracking of hydrocarbons, which is in line with the literature.^31^ Thus, we can conclude that the new setup is well suited to perform studies on plastic pyrolysis to BTX, giving BTX yields of 22.3 wt %. Operational issues were not encountered, and confirmed by performing experiments for longer TOS (*vide infra*).

### 3.3 Effects of Pyrolysis Temperature

The versatile design of the double-fluidized-bed reactor allows testing of a wide range of operating conditions, such as the temperatures of pyrolysis and aromatization reactors. Initial experiments were performed to investigate the effects of temperature of the pyrolysis reactor (500–700 °C) on BTX yields while keeping all other conditions at benchmark conditions (Table 2).

#### 3.3.1 Product Yields versus Temperature

**Figure 4:**
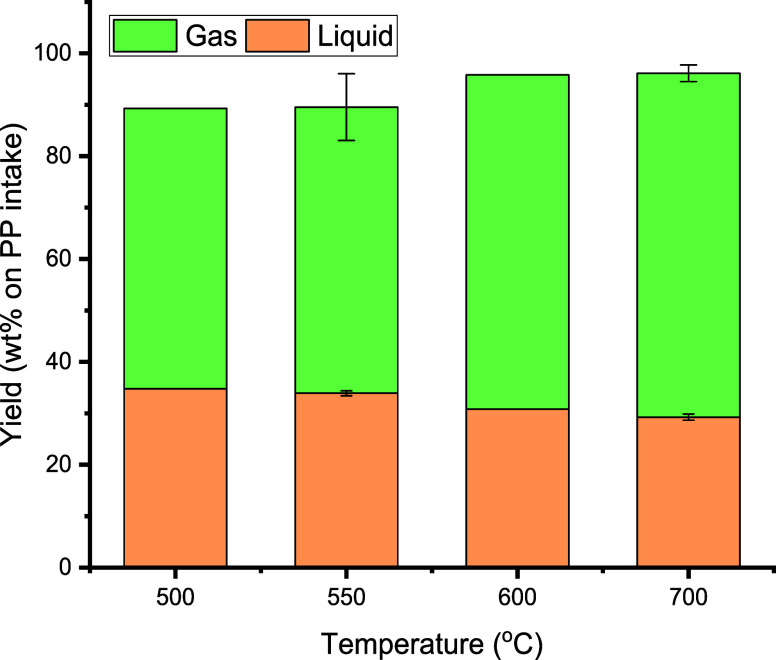
Effects of temperature in the first pyrolysis reactor on the gas and liquid yield (first pyrolysis reactor: 100 g of sand (45–100 μm), second aromatization reactor: 550 °C, 100 g of H-ZSM-5(38) catalyst (45–100 μm)).

The results of the experiments are shown in Figure 4. Upon increasing the pyrolysis temperature from 500 to 700 °C, the liquid yield reduced slightly from 34.8 to 29.3 wt %, Concomitantly, the gas yield increased from 54.4 to 66.9 wt % (Figure 4). Thus, higher pyrolysis temperatures lead to more excessive cracking and the formation of higher amounts of gas-phase products. This trend is consistent with findings reported for the catalytic pyrolysis of PP in the literature where the gas yield increased from 23 to 47 wt % when the temperature was increased from 600 to 1000 °C.^32^

#### 3.3.2 Liquid-Phase Composition versus Temperature

Our prime objective is high BTX yields, and as such, the liquid phases obtained at different pyrolysis temperatures were characterized in detail using GC-MS. Representative chromatograms (550 and 700 °C) are given in Figure S1 and Table S1.

**Figure 5:**
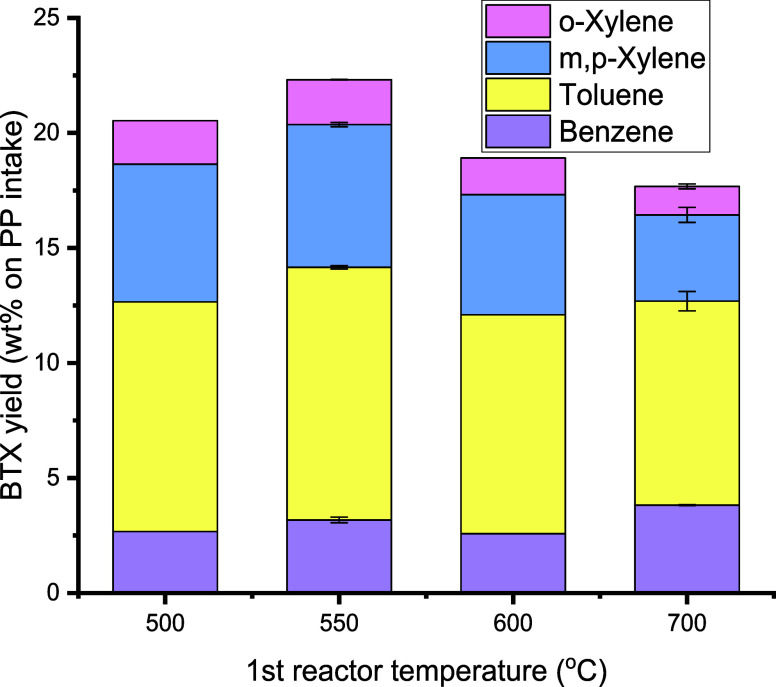
Effects of the first pyrolysis reactor temperature on BTX yield (conditions: first pyrolysis reactor: 100 g of sand (45–100 μm), second aromatization reactor: T2 = 550 °C, 100 g of H-ZSM-5(38) (45–100 μm), feed rate: 143 g/h PP, N_2_ flow: 0.66 L/min, TOS = 2 h).

The BTX yield shows a clear optimum at 550 °C of 22.3 wt % (on PP intake, Figure 5). Thus, while the amount of liquid product has reduced upon increasing the temperature from 500 to 700 °C (Figure 4), the BTX yield shows an optimum. Apparently, the vapor composition after the first pyrolysis step also affects the BTX yields (*vide infra*).

#### 3.3.3 BTX Selectivity versus Temperature

**Figure 6:**
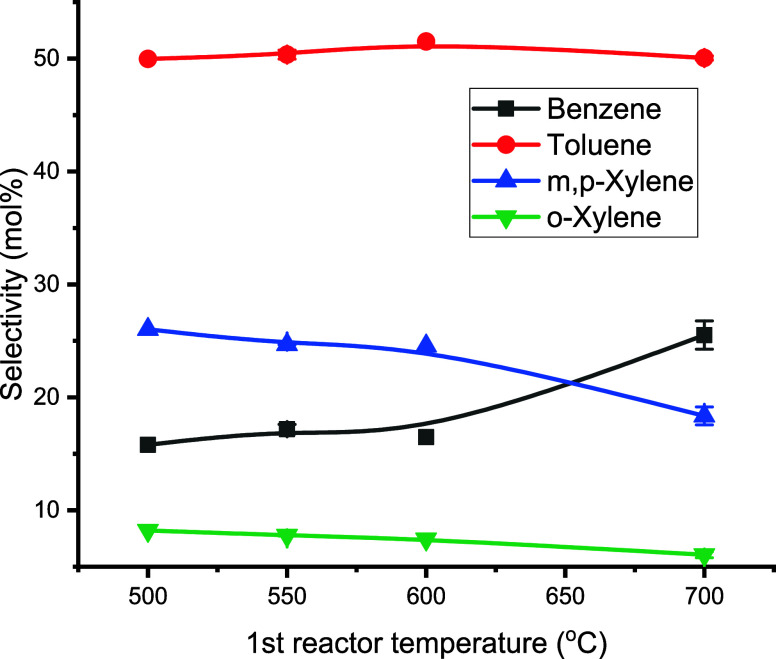
Effects of the first pyrolysis reactor temperature on the selectivity to individual BTX (conditions: first pyrolysis reactor: 100 g of sand (45–100 μm), second aromatization reactor: T2 = 550 °C, 100 g of H-ZSM-5(38) (45–100 μm), feed rate: 143 g/h PP, N_2_ flow: 0.66 L/min, TOS = 2 h).

Toluene is the most abundant component in the BTX fraction at all temperatures, with a selectivity of about 50 mol % (Figure 6). Nishino et al. reported a toluene selectivity of 44% for the catalytic pyrolysis of industrial plastic waste in a pilot plant.^33^ The pyrolysis temperature particularly affects benzene and xylene selectivities. At higher temperatures, the benzene selectivity increases considerably at the expense of xylene. A similar trend of selectivity as a function of temperature was also observed in the literature.^34^ For example, the benzene yield increased from 12 to 22% whereas the xylene yield decreased from 10 to 5% when increasing the temperature from 450 to 600 °C for the aromatization of *n*-butane. A possible explanation for this observation may be found when considering the gas-phase composition entering the aromatization reactor, which differs considerably for the two temperatures (*vide infra*). A more extended discussion is given in Section 3.4.

**Figure 7:**
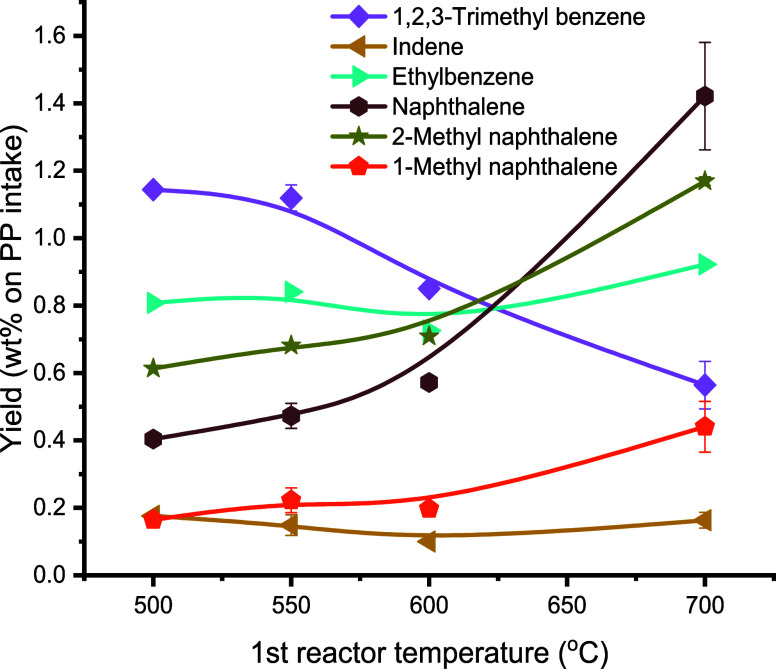
Effects of the first pyrolysis reactor temperature on the yield of other aromatics (conditions: first pyrolysis reactor: 100 g of sand (45–100 μm), second aromatization reactor: T2 = 550 °C, 100 g of H-ZSM-5(38) (45–100 μm), feed rate: 143 g/h PP, N_2_ flow: 0.66 L/min, TOS = 2 h).

The non-BTX aromatics were also quantified, and the yields are shown in Figure 7. At higher pyrolysis temperatures, the yield of 1,2,3-trimethylbenzene decreased, whereas those of ethylbenzene and naphthalene and its derivatives (i.e., 1-methyl naphthalene and 2-methyl naphthalene) increased significantly. The increase in the naphthalene yield from 0.4 to 1.4 wt % agrees with the reported trend in ref. (28) for the catalytic pyrolysis of PE using ZSM-5. It shows that the naphthalene yield increased 5-fold when increasing the pyrolysis temperature from 400 to 600 °C.

#### 3.3.4 Gas-Phase Composition

**Figure 8:**
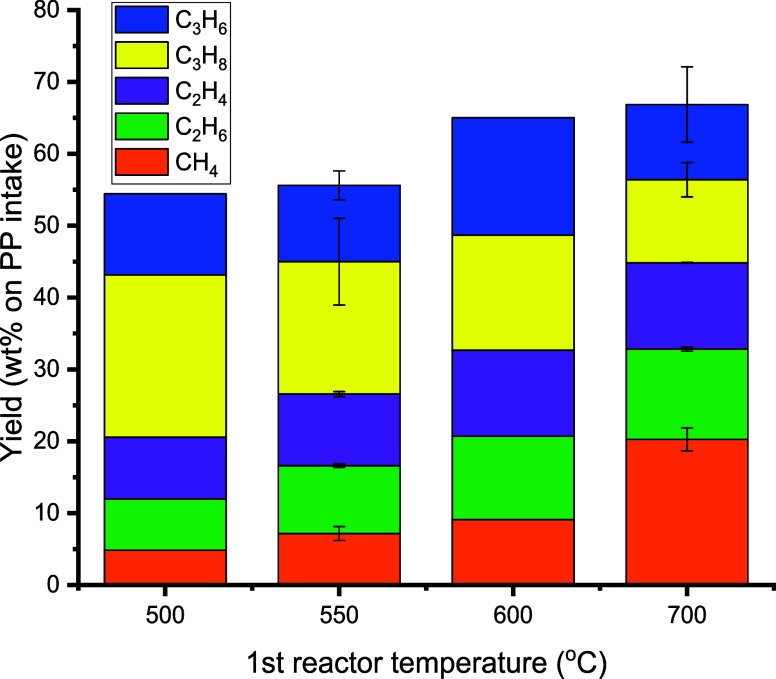
Effects of the first pyrolysis reactor temperature on the yield of gas-phase products (conditions: first pyrolysis reactor: 100 g of sand (45–100 μm), second aromatization reactor: T2 = 550 °C, 100 g of H-ZSM-5(38) (45–100 μm), feed rate: 143 g/h PP, N_2_ flow: 0.66 L/min, TOS = 2 h).

The gas-phase products in the outlet of the aromatization unit at different pyrolysis temperatures were quantified. The results are given in Figure 8. With increasing temperature, more methane, ethane, and ethene and less propane were formed, indicating a higher extent of cracking in the pyrolysis section, which agrees with a reported trend in ref. (28)

### 3.4 Noncatalytic Pyrolysis Experiments Using PP

**Figure 9:**
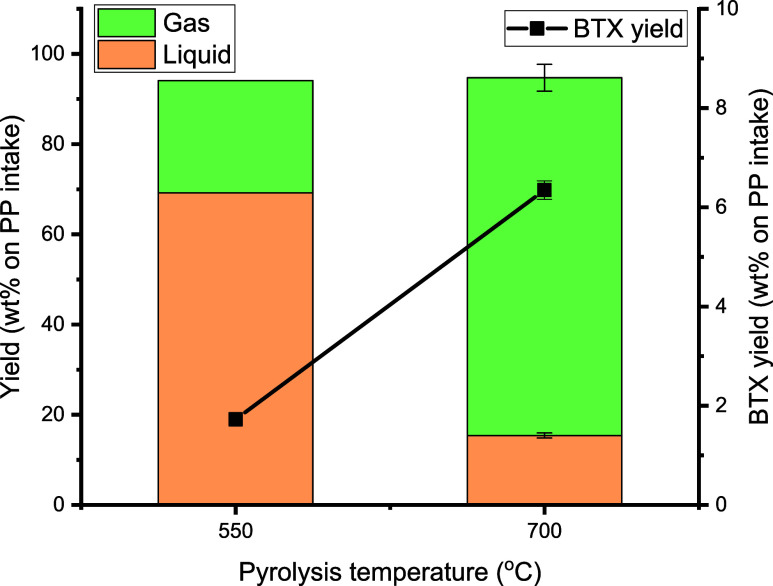
Effects of noncatalytic pyrolysis temperature on mass balance and BTX yield (feed: PP, first pyrolysis reactor: 100 g of sand (45–100 μm), second aromatization reactor: empty, N_2_ flow: 0.66 L/min, TOS = 2 h).

The effect of the pyrolysis reactor temperature on BTX yield may be explained by considering (i) the amount of vapor phase formed versus temperature in the first thermal pyrolysis reactor and (ii) the vapor composition in the exit of the first pyrolysis reactor, which is the input for the aromatization reactor. Information about the amount and composition of the vapor phase versus temperature was obtained by performing noncatalytic experiments at otherwise similar conditions. In this case, the aromatization reactor was empty and contained no catalyst. Two noncatalytic pyrolysis experiments were conducted at 550 and 700 °C. The results are shown in Figure 9.

**Figure 10:**
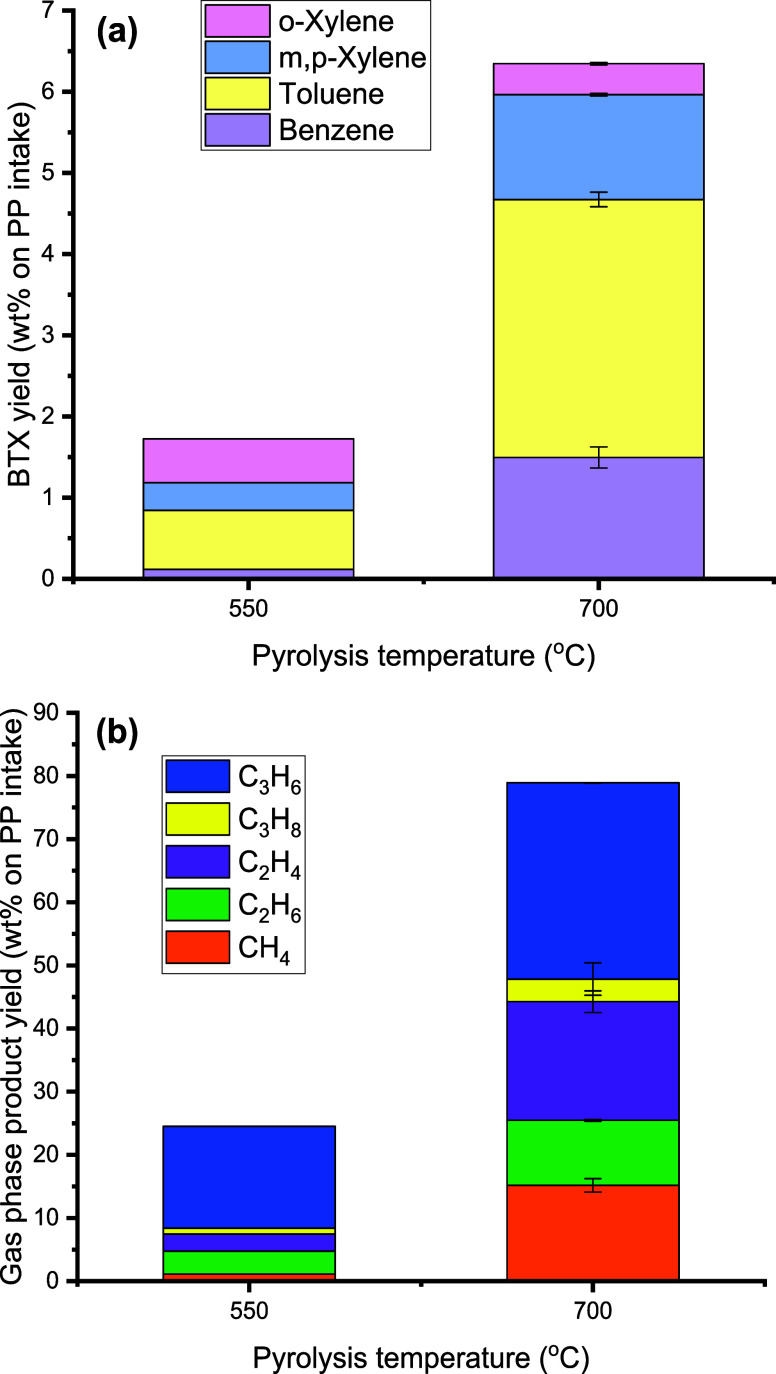
Effects of noncatalytic pyrolysis temperature on (a) BTX yield (wt % on PP intake) and (b) gas-phase product yield (wt % on PP intake) (feed: PP, first pyrolysis reactor: 100 g of sand (45–100 μm), second aromatization reactor: empty, N_2_ flow: 0.66 L/min, TOS = 2 h).

The amount of liquid phase reduced dramatically from 69 to 15 wt % when the temperature was increased from 550 to 700 °C in the pyrolysis section, and by far, more noncondensable gases were formed (Figure 9 and Table S2), indicating severe thermal cracking of the PP at elevated temperatures. Interestingly, higher temperature also led to higher thermal BTX yields. For instance, the BTX yield at 700 °C was 6.3 wt % (on PP intake) compared to only 1.7 wt % at 550 °C (Figure 10a). These findings of higher thermal BTX yields at elevated temperatures agree with the literature data for the noncatalytic pyrolysis of PP. For example, a BTX yield of 9.7 wt % was obtained from the noncatalytic pyrolysis of PP at 703 °C, though here the product gas was used as the fluidizing medium.^35^ Similarly, it is reported that BTX yields increased from 16.8% at 685 °C to 21.5% at 738 °C for the thermal pyrolysis of mixed plastics.^36^

The naphthalene yield for the noncatalytic experiments increased from 0.03% to 0.3% when the pyrolysis temperature was increased from 550 to 700 °C. Under catalytic conditions, the naphthalene yields were much higher and increased from 0.5 to 1.5% when the pyrolysis temperature increased from 550 to 700 °C. Thus, we can conclude that (i) the thermal naphthalene yield was by far less than the catalytic naphthalene yield and (ii) higher temperatures led in both cases to more naphthalenes. Thus, naphthalene was mainly produced in the second aromatization reactor by the action of the catalyst.

The gas-phase composition was highly dependent on the temperature; see Figure 10 for details. Propene was by far the major gas-phase component for the noncatalytic pyrolysis of PP at 550 °C. At 700 °C, the selectivity to propene was reduced considerably, and larger amounts of lower carbon number components were formed, such as ethylene, ethane, and methane, due to extensive cracking. This change in the gas-phase composition may also explain the observation that the selectivity of the individual components in the BTX fraction for catalytic pyrolysis was a function of the temperature (Figure 6). At 700 °C, the gas phase from the pyrolysis reactor was enriched in lower carbon number products compared to that at 500 °C. In particular, the amount of ethylene substantially increased; see Figure 10 for details. The relative reactivity for the catalytic pyrolysis of both ethylene and propene using an H-ZSM-5 catalyst at 500 °C has been studied in detail.^37^ It was reported that the BTX yield was independent of the olefin feed under the prevailing conditions, whereas the selectivity to benzene was substantially higher for ethylene than for propene. As such, the increase in the benzene selectivity at elevated temperatures may be due to the presence of higher amounts of ethylene in the feed to the aromatization reactor.

Detailed analyses of the liquid phase also indicate a higher extent of cracking at elevated temperatures. For example, TGA (Figure S2) reveals that the liquid product obtained at 700 °C was more volatile than the one at 550 °C.

In summary, the noncatalytic experiments imply that the observed effect of temperature on BTX yield in catalytic experiments was mainly due to changes in the composition of the vapor phase entering the aromatization reactor. At higher pyrolysis temperatures, the PP was more heavily cracked to low hydrocarbons and particularly those with a carbon number below 4. This appears to have a negative effect on the BTX yield, most likely due to the presence of significant amounts of methane in the product gas at high temperatures. The latter is known to be less prone to aromatization than the C2 and C3 hydrocarbons.^37^ The reduction in BTX yields at higher temperatures was partly mitigated by the observation that the thermal pyrolysis of PP at 700 °C gave a significant amount of BTX.

### 3.5 Effects of N_2_ Flow Rate on BTX Yields

The N_2_ flow rate and thus the superficial velocity in both reactors are expected to affect the fluidization regime and the average residence times of vapors. To investigate the effect of this process variable on the BTX yield, several experiments were performed at benchmark conditions (Table 2) while varying the N_2_ flow rate between 0.66 and 1.98 NL/min.

**Figure 11:**
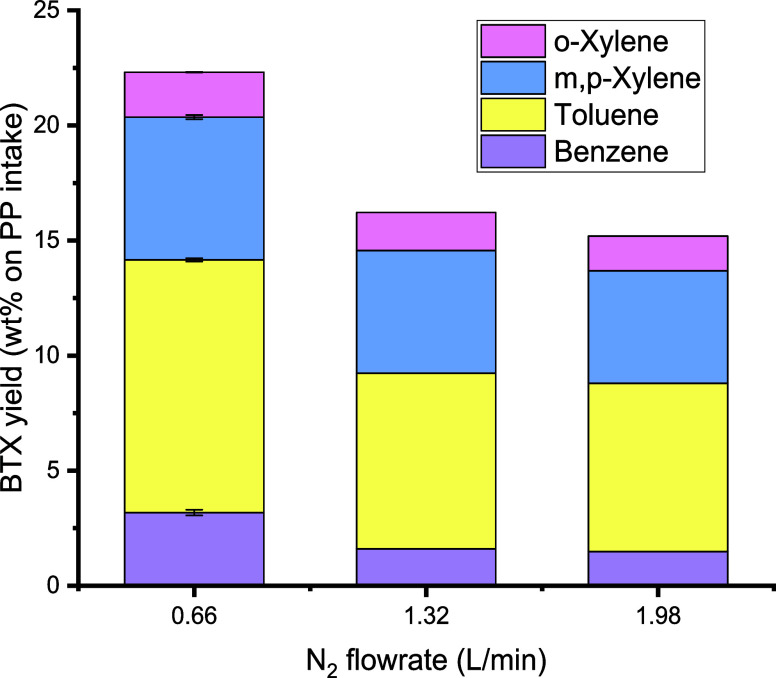
Effects of N_2_ flow rate on BTX yield (wt % on PP intake) (all reactor temperatures: 550 °C, first pyrolysis reactor: 100 g of sand (45–100 μm), second aromatization reactor: 100 g of H-ZSM-5(38) catalyst (45–100 μm)).

The effects of N_2_ flow rate on the gas and liquid yields are shown in Figure S3. The liquid yield reduced slightly from 32 to 28 wt % when the N_2_ flow rate was tripled. In addition, a significant effect of the flow rate on the BTX yield was detected; see Figure 11 for details. The BTX yield dropped from 22.3 wt % at the lowest flow rate to 15.2 wt % at the highest flow rate in the range. This agrees with the reported results for *in situ* batch catalytic pyrolysis of post consumer plastic waste using H-ZSM-5, showing that lower flow rates lead to higher BTX yields.^38^ The fluidization regime for both the pyrolysis and aromatization reactors was calculated using appropriate theoretical models and, in some cases, validated by experiments. This information is given in Section S1. The pyrolysis reactor was operated in the bubbling regime for a full range of flow rates. Calculation of the regime in the aromatization reactor was less straightforward, as significant amounts of vapor from the thermal PP pyrolysis entered the aromatization reactor and led to higher superficial velocities than when using nitrogen gas only. When compensating for this effect, the fluidization regime changed from bubbling at the lowest flow rate to slug flow at the two higher flow rates. Apparently, operation in the slug flow regime in the second aromatization reactor has a negative effect on the BTX yield.

An explanation for this observation may be the differences in the heat and mass transfer characteristics between the slug flow and bubbling flow fluidization regimes. However, more extensive studies, including CFD modeling, will be required to rationalize the observed experimental trends. These studies are ongoing, and the findings will be shared in due course.

### 3.6 Effects of Catalyst Particle Size on BTX Yield

The particle size of H-ZSM-5 is known to have a significant effect on the BTX yield in the pyrolysis of plastics. Smaller H-ZSM-5 particles tend to increase the yield of BTX due to a higher surface area, which enhances the catalyst’s activity by providing more active sites for the conversion of plastic pyrolysis vapors into aromatic compounds.^39^ However, the use of small particles may lead to operational challenges. Thus, optimizing the particle size of H-ZSM-5 is crucial to maximize BTX yields while ensuring process efficiency.

**Figure 12:**
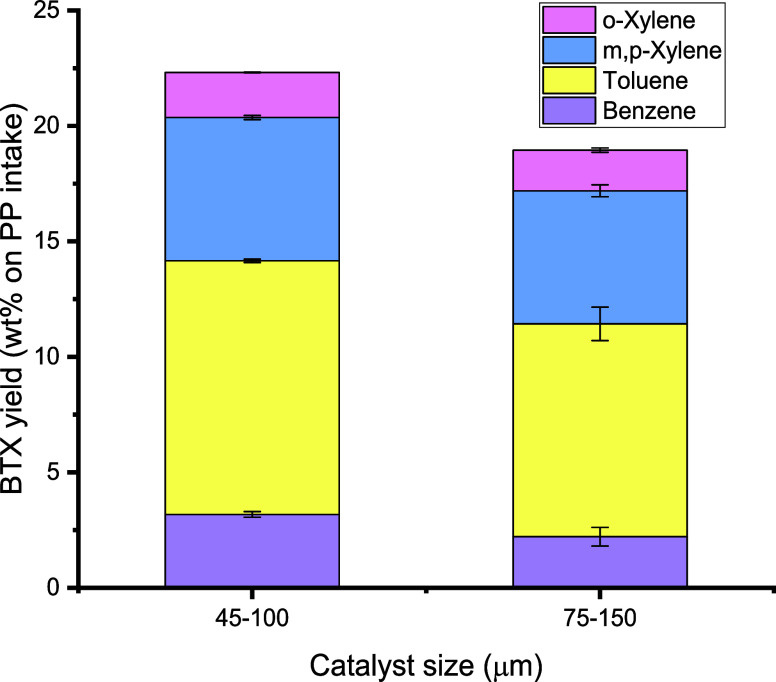
Effects of catalyst size on BTX yield (conditions: all reactor temperatures: 550 °C, first pyrolysis reactor: 100 g of sand (45–100 μm), second aromatization reactor: 100 g of H-ZSM-5(38) catalyst, N_2_ flow: 0.66 L/min).

The effects of particle size on the BTX yield in our double-fluidized-bed setup were investigated using two sieve fractions of the H-ZSM-5 catalyst (45–100 and 75–150 μm). All other conditions are given in Table 2 (benchmark). The effects of particle size on the liquid and gas yields are shown in Figure S4 and show no significant effect. The effects of particle size on the BTX yield are given in Figure 12. The BTX yield was considerably higher when using the smaller catalyst particles. This finding may be explained by considering possible changes in the fluidization regime when using different catalyst particle sizes in the aromatization reactor and/or effects on mass- and heat transfer rates. Calculations indicate that the fluidization regime for both catalyst sieve fractions was equal and that they were in the bubbling regime (see the Supporting Information). As such, the observed changes were not majorly due to fluidization effects. To determine whether mass transfer plays a role, the Thiele moduli and effectiveness factors were evaluated. The results are provided in Section S2. It can be concluded that the observed reaction rates and, thus, the BTX yields were determined by both the intrinsic chemical reaction rate and internal particle diffusion. The effect of internal particle diffusion was higher for the catalyst of larger size (Figure S5). As such, the higher yield of BTX for the smaller catalyst particles was most likely due to a reduction of intraparticle mass transfer limitations of reactants and products.

The catalyst particle size also had a small effect on the chemoselectivity of the reaction, and more xylenes were produced when using larger particles (Figure S6, 35.6 mol % for larger particles and 32.5 mol % for small particles). Most likely, mass transfer effects were responsible for this observation. It is possible that alkylation–dealkylation reactions to form xylenes occurred mainly in the micropores of the zeolite and that longer diffusional pathways enhanced the selectivity of xylenes. The literature also reports a higher para-xylene selectivity for toluene alkylation when using larger catalyst particles.^40^

### 3.7 Operational and Catalyst Stability

**Figure 13:**
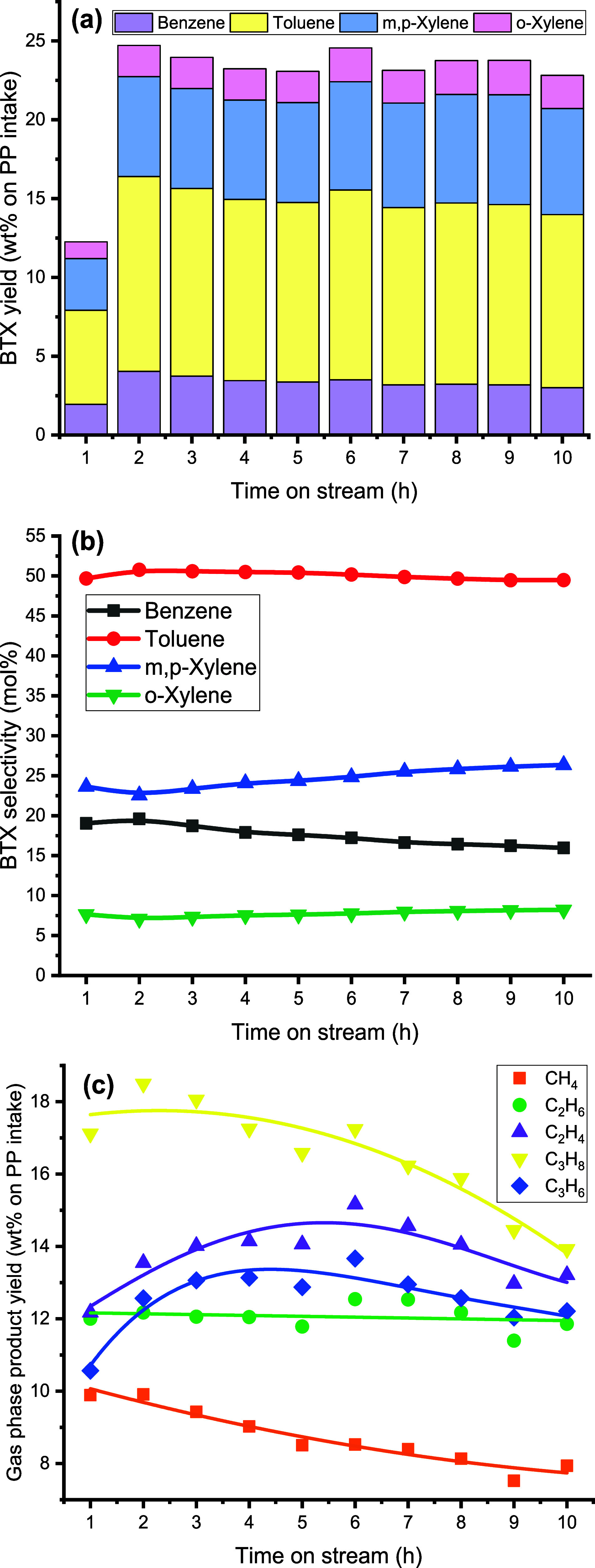
BTX yield (wt % on PP intake) (a), selectivity to individual BTX (mol %) (b), and gas-phase product yield (wt % on PP intake) (c), as a function of TOS (all reactor temperatures: 550 °C, first pyrolysis reactor: 100 g of sand (45–100 μm), second aromatization reactor: 100 g of catalyst (45–100 μm), N_2_ flow: 0.66 L/min).

To examine both operational and catalyst stability, an experiment at an extended TOS of 10 h was performed (benchmark conditions, Table 2). Operational issues were not encountered and a total amount of 1.5 kg of PP was fed into the unit over 10 h. The products were collected every hour, gravimetrically measured and analyzed. The results are given in Figures 13 and S7. The gas and liquid yields were relatively stable for a TOS of 10 h. The average liquid yield was 34.9 wt % on PP intake, which agrees well with the results for the 2 h TOS experiment discussed earlier. The BTX yield was low in the first hour, most likely due to the startup of the reactor. It reached 24.7 wt % after 2 h and then slowly decreased to a value of 22.8 wt % after 10 h (Figure 13a). Thus, we can conclude that catalyst stability was good and showed only a small decrease after 10 h. Of interest is the observation that the selectivity to toluene and benzene decreased whereas that to xylenes increased with longer TOS (Figure 13b). This gradual change in the selectivity to individual BTX components may be caused by coke formation on the zeolite catalyst (*vide infra*). This would have lowered the size of zeolite channels where the aromatization of reactive olefin species occurred and thus altered the selectivity of individual BTX compounds.

In addition, as shown in Figure 13c, the gas-phase composition was also a function of the TOS. For instance, the yields of CH_4_ and C_3_H_8_ dropped by 1.6 and 2.5 wt %, respectively. This also indicates the occurrence of changes in the catalyst structure at prolonged TOS.

### 3.8 Catalyst Characterization

To gain insight into possible changes in the catalyst structure after a TOS of 10 h, both the fresh and spent catalysts were characterized (TGA, N_2_-physisorption, and NH_3_-TPD). The TGA and DTGA curves are given in Figure S8. Based on the data, the coke content on the catalyst after a TOS of 10 h was 7.3 wt %. A clear DTGA peak is present at 583 °C and this classifies the coke as a hard coke.^41^ Though the amount of coke only has a minor impact on catalyst activity and the corresponding BTX yield (Figure 13), its formation is most likely the reason for changes in the BTX selectivity and gas-phase composition. Coke formation is due to oligomerization of reactive intermediates in the hydrocarbon pool at the catalyst surface during catalytic aromatization.

Coke formation also affects the acidity and BET surface area (Table 4 and Figures S9, S10). The surface area was reduced by 23% (from 342 to 264 m^2^/g), whereas the acidity was reduced by 24% after 10 h on stream. The NH_3_-TPD profile shows that the amounts of weak (centered at 200 °C) and strong acidic sites (centered at 380 °C) decrease by 19 and 51% (quantified by deconvolution), respectively, as a result of coke formation (Figure S9).^20^ Thus, we can conclude that the changes in the catalyst structure during aromatization are due to coke formation and a concomitant loss of acidity and surface area. However, this does not have a major effect on BTX yields. Apparently, more severe coking is required to make the catalysts less or even more inactive. Coke formation is not necessarily a showpiece for further commercialization of the technology. Regeneration protocols, e.g., by oxidative treatments have been developed for coked zeolite catalysts, though regeneration is often more cumbersome when dealing with hard cokes.^20,41^

**Table 4:** Characterization of Fresh and Spent Catalysts

	BET surface area (m^2^/g)	pore volume (cm^3^/g)	TPD total acidity (μmol/g)
fresh	342	0.396	548
TOS = 10 h	264	0.281	415

## 4 Conclusions

In this article, we have demonstrated that a double-fluidized-bed reactor is highly efficient for the *ex situ* catalytic aromatization of plastic waste (PP) to BTX. Operability was excellent for a runtime of 10 h. It allows for flexibility in process conditions, and for instance the reactor temperatures may be tuned independently. The effects of pyrolysis temperature in the thermal pyrolysis reactor on BTX yield were investigated and the optimal temperature was 550 °C, affording a BTX yield of 22.3 wt % based on PP intake. Thermal pyrolysis studies at similar conditions show that the extent of cracking is particularly enhanced at higher temperatures, leading to significant amounts of small hydrocarbon gases and mainly methane. The latter was more difficult to aromatize to BTX in the second catalytic aromatization reactor, explaining the lower BTX yields at higher temperatures. This effect was counteracted by the observation that the thermal BTX yields increased with temperature (6.3 wt % at 700 °C versus 1.7 wt % at 550 °C). The effects of the fluidization gas flow rate and catalyst particle size were also explored and revealed that higher BTX yields were attainable at a lower N_2_ flow rate and smaller particle sizes. Experiments at extended times on stream (10 h) showed that the BTX yield is only slightly reduced, most likely due to coke formation and concomitant reductions in the BET surface area and catalyst acidity. The concept of a double fluidized bed is currently being scaled up to a pilot scale by the company BIOBTX.

## Supporting Information Available

Determination of flow regime; GC-MS chromatogram analysis of liquid products; liquid distillation profile by TGA; mass balance and selectivity analysis; Thiele modulus and effectiveness factor calculations; TGA and DTG of the spent catalyst; and NH_3_-TPD profiles and nitrogen adsorption/desorption isotherms of fresh and spent catalysts (PDF)
