# Supplementary material for: Catalytic Pyrolysis of Polypropylene to Benzene, Toluene, and Xylene (BTX) Using a Double-Fluidized-Bed Reactor
Source: Energy Fuels. 2025 Feb 7;39(7):3564–74. doi: 10.1021/acs.energyfuels.4c05316 (PMC11849447; doi:10.1021/acs.energyfuels.4c05316)
Supplement: Supplementary file 1 — ef4c05316_si_001.pdf [file ef4c05316_si_001.pdf]

Supporting information for:

Catalytic pyrolysis of polypropylene to benzene, toluene and xylene (BTX) using a double fluidized bed reactor

Hongqi Wang[a], Matthijs van Akker[b], Jozef G. M. Winkelman[a], André Heeres[c], Hero Jan Heeres[a,*]

[a] Green Chemical Reaction Engineering, Engineering and Technology Institute Groningen, University of Groningen, Nijenborgh 4, 9747 AG, Groningen, the Netherlands

[b] BioBTX BV, Zernikelaan 17, 9747 AA, Groningen, the Netherlands

[c] Hanze University of Applied Sciences, Zernikeplein 11, 9747 AS, Groningen, the Netherlands

* Corresponding author.
E-mail address: h.j.heeres@rug.nl (H.J. Heeres).

Section S1. Determination of flow regime

S1.1 Constants and properties

Nitrogen density at 550 ^{o}C = 0.409 kg/m^3

Nitrogen viscosity at 550 ^{o}C = 36.59 $N\ s/m^2 \times 10^{-6}$

Gravitational acceleration = 9.8 m/s^2

S1.2 Definitions

Archimedes Number:

$$A_r = \frac{\rho_G(\rho_P - \rho_G)gd_p^3}{\mu_G^2} = (d_p^*)^3$$

where ρ_G and ρ_P are the gas and particle density, μ_G is the gas viscosity and g is the acceleration of gravity.

1) Minimum fluidization velocity [1]

$$U_{mf} = \frac{\mu}{\rho_f d_p}[\sqrt{33.7^2 + 0.0408Ar} - 33.7]$$

2) Minimum bubbling velocity [2]

$$U_{mb} = 33d_p\left(\frac{\rho_G}{\mu_G}\right)^{0.1}$$

3) U_{ms} minimum gas superficial velocity for slug flow [3]

$$U_{ms} = U_{mf} + 0.07(gD)^{0.5}$$

where *D* is the column diameter (m).

(4) Uc: onset superficial gas velocity for turbulent fluidization [3]

$$Re_c = \frac{\rho_g U_c d_p}{\mu_g} = 0.565Ar^{0.461}$$

1st fluidized bed reactor: sand (45-100 μm)

Average diameter of sand = 72.5 ± 27.5 μm

When mass flow of 1 L/min at 25 °C is heated to 550 °C, the corresponding superficial velocity U is 0.023 m/s =2.3 cm/s at 550 °C. At 550 °C, 0.66, 1.32 and 1.98 L/min N_2 have the superficial velocity of 1.6, 3.1 and 4.6 cm/s, respectively.

Archimedes number at 550 °C:

$$Ar = \frac{0.409 \cdot (2400 - 0.409) \cdot 9.8 \cdot \left(\left(72.5 \cdot 10^{-6} \right)^3 \right)}{\left(36.59 \cdot 10^{-6} \right)^2} = 2.74$$

1) Minimum fluidization velocity

$$U_{mf} = \frac{36.59 \cdot 10^{-6}}{0.409 \cdot \left(72.5 \cdot 10^{-6} \right)} \cdot \left(\sqrt{\left(33.7^2 + 0.0408 \cdot 2.737636665 \right)} - 33.7 \right) = 0.002 \text{ m/s} = 0.2 \text{ cm/s}$$

The corresponding N_2 flowrate is 0.1 L/min.

Based on previous studies in our group, the experimentally determined flow rate for 100-212 µm sand at 550 °C corresponding to the minimum fluidization velocity is 0.6 L/min as shown in Figure S1 [4]. In this study, the sand particle diameter was reduced by half to 45-100 µm and the corresponding flow rate should be reduced by fourfold in theory with the value being 0.15 L/min, which is close to the above calculated 0.1 L/min.

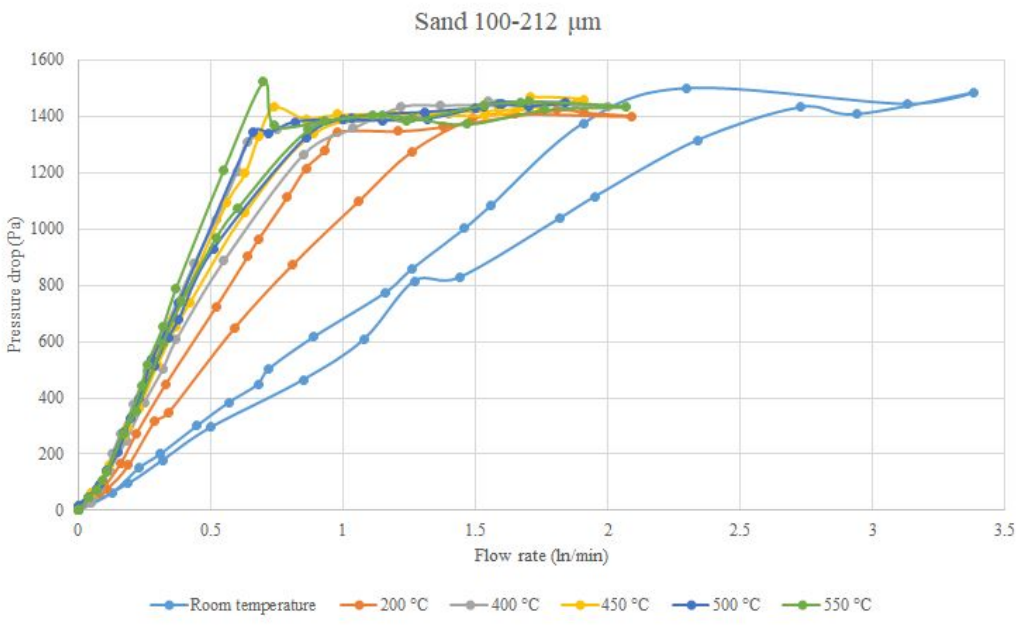

Figure S1. Pressure drop in the fluidized bed reactor using sand (100-212 µm) at different normal flow rate [4].

2) Minimum bubbling velocity

$$U_{mb} = 33 \cdot \left(72.5 \cdot 10^{-6}\right) \cdot \left(\frac{0.409}{36.59 \cdot 10^{-6}}\right)^{0.1} = 0.006 \text{ m/s} = 0.6 \text{ cm/s}$$

3) *U_{ms}* minimum gas superficial velocity for slug flow

$$U_{ms} = 0.002044881555 + 0.07 \cdot \left(\frac{9.8 \cdot 5}{100}\right)^{0.5} = 0.051 \text{ m/s} = 5.1 \text{ cm/s}$$

2nd fluidized bed reactor

Assume the reaction in 1st reactor is: $C_{30}H_{60} = 10.5\ C_2H_4 + 3\ C_3H_6$

The amount of vapour from 1st pyrolysis reactor to the 2nd aromatization reactor was estimated by Aspen Plus. For 0.66 L/min, the amount of pyrolysis vapor to 2nd reactor is 6.12 L/min or 0.000102 m^3/s at 550 ^oC, the superficial velocity calculated by the vapour volume is 5.2 cm/s. The simulated superficial velocity distribution is 5.3 cm/s at bottom and 2.1 cm/s at height >26 cm. Similarly, for 1.32 L/min, the amount of pyrolysis vapor to 2nd reactor is 7.903 L/min at 550 ^oC. The superficial velocity calculated by the vapour volume is 6.7 cm/s. The simulated superficial velocity distribution is 6.9 cm/s at bottom and 2.7 cm/s at height > 26 cm. For 1.98 L/min, the amount is pyrolysis vapor to 2nd reactor is 9.66 L/min at 550 ^oC, and the superficial velocity calculated by the vapour volume is 8.2 cm/s. The simulated superficial velocity distribution is 4.1 cm/s at bottom, 6.1 cm/s at height = 20 cm, and 2.7 cm/s at height >26 cm.

2nd fluidized bed reactor: for H-ZSM-5 catalyst of 75-150 µm

The average size is 112.5 ± 37.5 µm. Ar = 10.22867445.

1) Minimum fluidization velocity

$$U_{mf} = \frac{36.59 \cdot 10^{-6}}{0.409 \cdot \left(112.5 \cdot 10^{-6}\right)} \cdot \left(\sqrt{\left(33.7^2 + 0.0408 \cdot 10.22867445\right)} - 33.7\right) = 0.0049 \text{ m/s} = 0.49 \text{ cm/s}$$

2) Minimum bubbling velocity

$$U_{mb} = 33 \cdot (112.5 \cdot 10^{-6}) \cdot \left(\frac{0.409}{36.59 \cdot 10^{-6}} \right)^{0.1}$$ =0.009 m/s =0.9 cm/s

3) U_{ms} minimum gas superficial velocity for slug flow

$$U_{ms} = 0.004923413623 + 0.07 \cdot \left(\frac{9.8 \cdot 5}{100} \right)^{0.5}$$ =0.054 m/s =5.4 cm/s

(4) U_c: onset superficial gas velocity for turbulent fluidization

$$U_c = \frac{0.565 \cdot 10.22867445^{0.461} \cdot 36.59 \cdot 10^{-6}}{0.409 \cdot (112.5 \cdot 10^{-6})}$$ =1.31 m/s = 131 cm/s

2nd fluidized bed reactor: for H-ZSM-5 catalyst of 45-100 µm

The average size is 72.5 ± 27.5 µm. Ar = 2.737636665

1) Minimum fluidization velocity

$$U_{mf} = \frac{36.59 \cdot 10^{-6}}{0.409 \cdot (72.5 \cdot 10^{-6})} \cdot \left(\sqrt{(33.7^2 + 0.0408 \cdot 2.737636665)} - 33.7 \right)$$ =0.002 m/s= 0.2 cm/s

2) Minimum bubbling velocity

$$U_{mb} = 33 \cdot (72.5 \cdot 10^{-6}) \cdot \left(\frac{0.409}{36.59 \cdot 10^{-6}} \right)^{0.1}$$ =0.0061 m/s =0.61 cm/s

3) U_{ms} minimum gas superficial velocity for slug flow

$$U_{ms} = 0.002044881555 + 0.07 \cdot \left(\frac{9.8 \cdot 5}{100} \right)^{0.5}$$ =0.051 m/s =5.1 cm/s

(4) Uc: onset superficial gas velocity for turbulent fluidization

$$U_c = \frac{0.565 \cdot 2.737636665^{0.461} \cdot 36.59 \cdot 10^{-6}}{0.409 \cdot (72.5 \cdot 10^{-6})}$$ =1.109 m/s = 111 cm/s

Summary of flow regime for the double fluidized bed reactor at 550 ^{o}C

	Particle type and size	Minimum fluidization velocity (cm/s) **U_{mf}**	Minimum bubbling velocity(cm/s) **U_b**	Minimum superficial velocity for slug flow **U_{ms}**	Onset superficial velocity for turbulent fluidization **U_c**	N_2 flowrate (L/min)	Superficial velocity (cm/s); assuming reaction of C30H60 using Aspen plus for 2nd reactor	Fluidization regime
1st reactor	Sand, 45-100 um	0.2	0.6	5.1		0.66	1.6	Bubbling
1st reactor	Sand, 45-100 um	0.2	0.6	5.1		1.32	3.1	Bubbling
1st reactor	Sand, 45-100 um	0.2	0.6	5.1		1.98	4.6	Bubbling
2nd reactor	H-ZSM-5, 75 – 150 µm	0.49	0.9	5.4	131	0.66	5.2	Bubbling
						1.32	6.7	Slugging
2nd reactor	H-ZSM-5, 45 – 100 µm	0.2	0.61	5.1	111	0.66	5.2 (2.1 – 5.3, aspen)	Bubbling
						1.32	6.7 (2.7 – 6.9, aspen)	Slugging
						1.98	8.2 (4.1 bottom, 6.1 at height =20, 2.7 at h >26cm from aspen)	Slugging

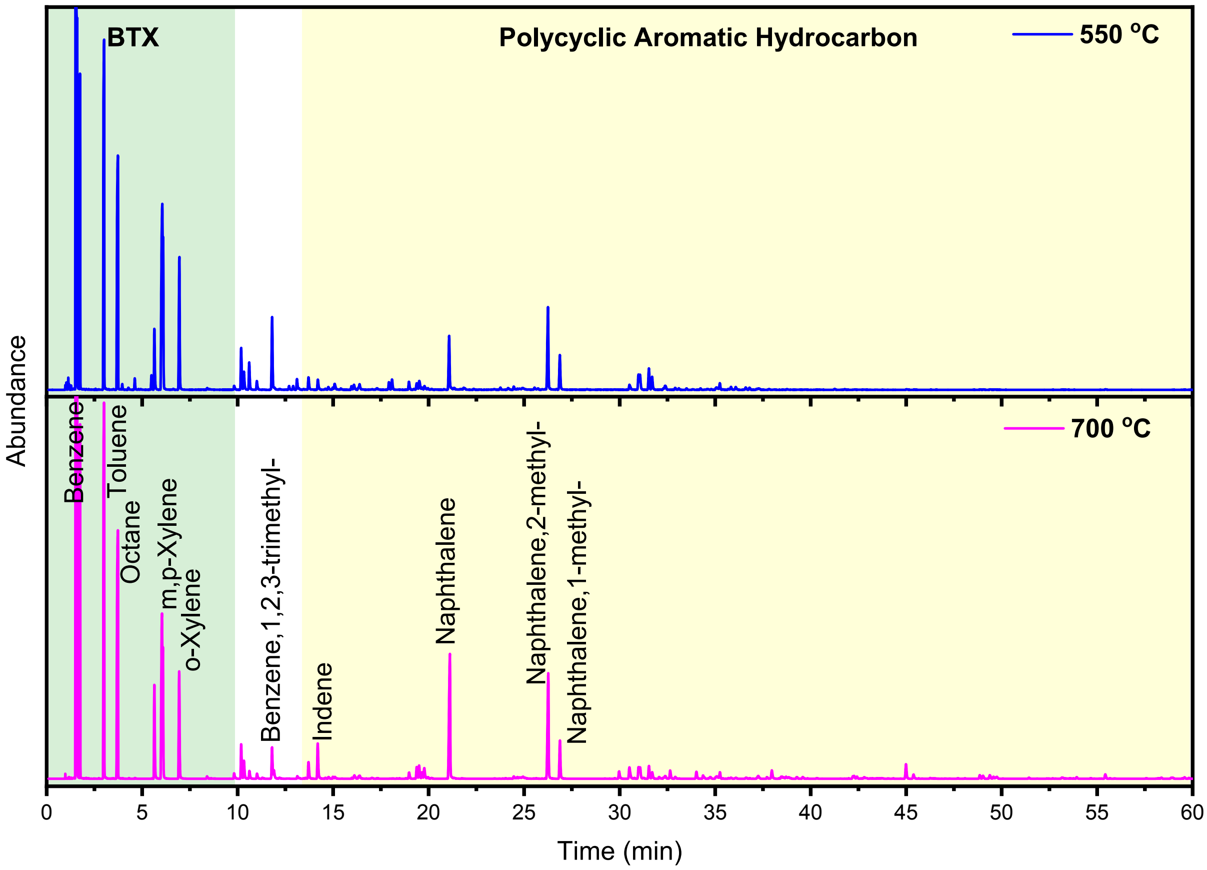

Figure S1. Effects of pyrolysis temperature on the GC-MS of the liquid products (1st pyrolysis reactor: 100 g sand (45-100 μm), 2nd aromatization reactor: 550 °C, 100 g H-ZSM-5(38) catalyst (45-100 μm)).

Table S1. GC-MS chromatogram analysis of catalytic pyrolysis (Figure 5) at 550 ^{o}C (The structures were determined by comparing with NIST98 library).

Time (min)	Name	Formula	Structure
1.747	Benzene	C6H6	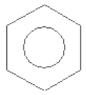
3.001	Toluene	C7H8	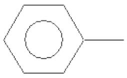
3.956	Cyclopentane,1,1,3,4-tetramethyl-,cis-	C9H18	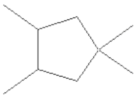
4.285	Heptane,2,4-dimethyl-	C9H20	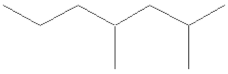
4.613	Cyclohexane,1,3,5-trimethyl-	C9H18	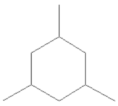
5.485	Cyclohexane,1,2,4-trimethyl-	C9H18	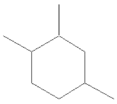
5.639	Ethylbenzene	C8H10	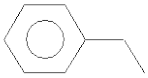
6.046	p-Xylene	C8H10	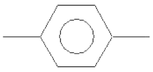
6.931	o-Xylene	C8H10	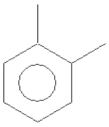
8.399	Benzene,(1-methylethyl)-	C9H12	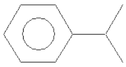
9.815	Benzene,propyl-	C9H12	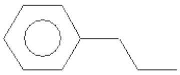
10.183	Benzene,1-ethyl-3-methyl-	C9H12	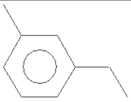
10.323	Benzene,1-ethyl-4-methyl-	C9H12	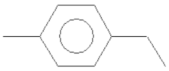
10.604	Benzene,1,2,3-trimethyl-	C9H12	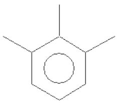

11.002	Benzene,1-ethyl-2-methyl	C_9H_{12}	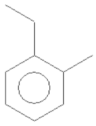
11.804	Benzene,1,3,5-trimethyl-	C_9H_{12}	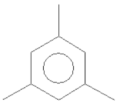
12.694	Heptane,5-ethyl-2-methyl-	$C_{10}H_{22}$	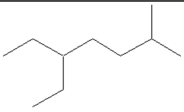
12.900	Nonane,2,6-dimethyl-	$C_{11}H_{24}$	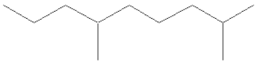
13.711	Indane	C_9H_{10}	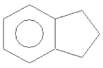
14.197	Indene	C_9H_8	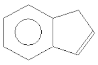
14.596	Benzene,1,3-diethyl-	$C_{10}H_{14}$	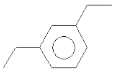
14.745	Benzene,1-methyl-3-propyl-	$C_{10}H_{14}$	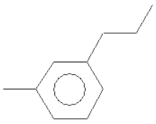
14.981	Benzene,1-methyl-2-propyl-	$C_{10}H_{14}$	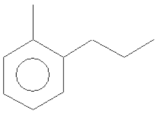
15.082	Benzene,1,2,3,5-tetramethyl-	$C_{10}H_{14}$	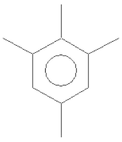
15.398	Dodecane	$C_{12}H_{26}$	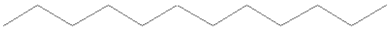
15.959	Benzene,2-ethyl-1,4-dimethyl-	$C_{10}H_{14}$	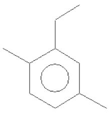
16.086	Benzene,4-ethyl-1,2-dimethyl-	$C_{10}H_{14}$	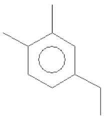
17.304	Benzene,1-ethyl-2,3-dimethyl	$C_{10}H_{14}$	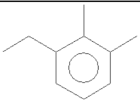
17.922	Benzene,1,2,4,5-tetramethyl-	$C_{10}H_{14}$	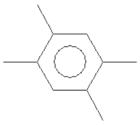
18.084	Benzene,1,2,3,4-tetramethyl-	$C_{10}H_{14}$	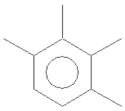

18.969	1H-Indene,2,3-dihydro-4-methyl-	C10H12	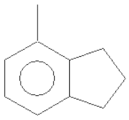
19.236	Benzene,1,3-diethyl-5-methyl-	C11H16	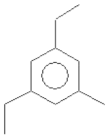
19.372	1H-Indene,3-methyl-	C10H10	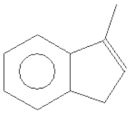
19.508	Naphthalene,1,2-dihydro-	C10H10	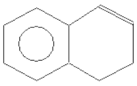
19.661	2-Methylindene	C10H10	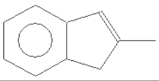
19.780	1H-Indene,1-methyl-	C10H10	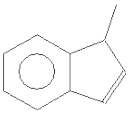
19.968	Naphthalene,1,2,3,4-tetrahydro-	C10H12	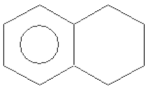
21.068	Naphthalene	C10H8	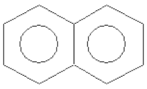
26.239	Naphthalene,2-methyl-	C11H10	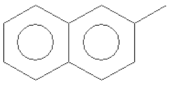
26.874	Naphthalene,1-methyl-	C11H10	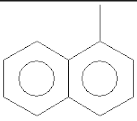
30.516	Naphthalene,1-ethyl-	C12H12	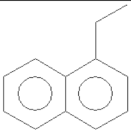
30.989	Naphthalene,1,5-dimethyl-	C12H12	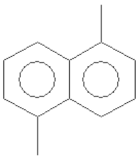
31.528	Naphthalene,1,7-dimethyl-	C12H12	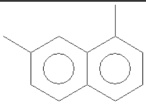
31.704	Naphthalene,2,3-dimethyl-	C12H12	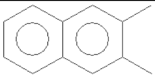
32.392	Naphthalene,1,8-dimethyl-	C12H12	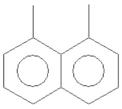

32.913	Naphthalene,1,4-dimethyl-	C12H12	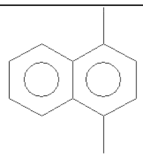
33.093	Dodecane,4,6-dimethyl-	C14H30	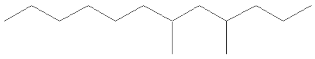
33.500	Decane,2,3,5,8-tetramethyl-	C14H30	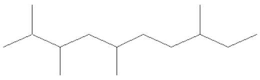
34.241	Heptadecane	C17H36	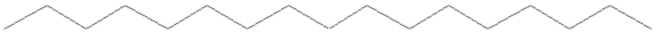
34.793	Naphthalene,2-(1-methylethyl)-	C13H14	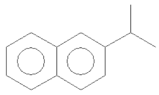
35.069	Naphthalene,1,4,6-trimethyl-	C13H14	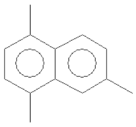
35.827	Naphthalene,2,3,6-trimethyl-	C13H14	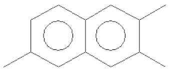
36.081	Naphthalene,1,6,7-trimethyl-	C13H14	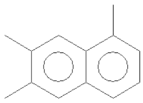
36.603	Naphthalene,1,4,5-trimethyl-	C13H14	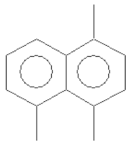
40.428	Naphthalene,1,2,3,4-tetramethyl-	C14H16	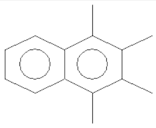
41.261	Naphthaleme,1-methyl-7-(1-methylethyl)-	C14H16	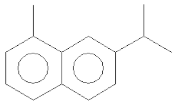
45.034	Anthracene	C14H10	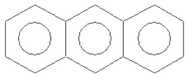
48.873	Phenanthrene,4-methyl-	C15H12	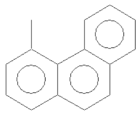
49.057	Anthracene,2-methyl-	C15H12	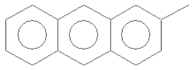
49.390	Phenanthrene,3-methyl-	C15H12	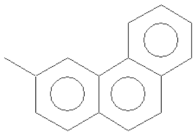
49.591	1H-Indene,3-phenyl-	C15H12	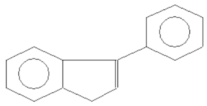

23.167	Phenanthrene,3,6-dimethyl-	C16H14	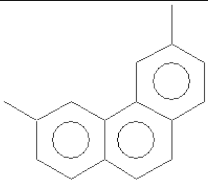
55.433	Pyrene	C16H10	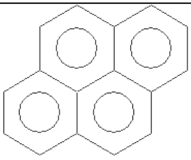
58.974	7H-Benzo[c]fluorene	C17H12	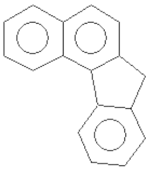
59.819	11H-Benzo[a]fluorene	C17H12	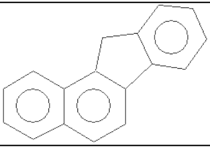

Table S2. GC-MS chromatogram analysis of noncatalytic pyrolysis (Figure 11a) at 550 ^{o}C (The structures were determined by comparing with NIST98 library).

Time (min)	Name	Formula	Structure
1.539	Pentane	C5H12	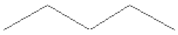
1.833	1-Pentene,2-methyl-	C6H12	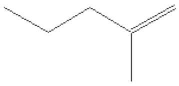
2.302	2,4-Dimethyl 1,4-pentadiene	C7H12	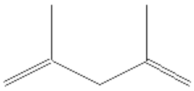
2.823	1,3-Pentadiene,2,3-dimethyl-	C7H12	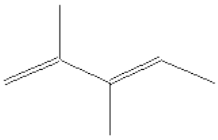
3.498	Trans,trans-1,3,5-Heptatriene	C7H10	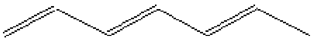
3.805	3-Heptene,4-methyl-	C8H16	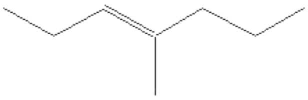
4.029	Heptane,4-methyl-	C8H18	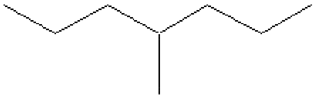
4.213	1,5-Hexadiene,2,5-dimethyl-	C8H14	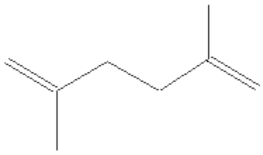
6.202	2-Heptene,2,3-dimethyl-	C9H18	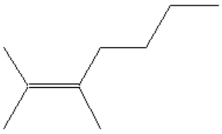
6.553	2,4-Dimethyl-1-heptene	C9H18	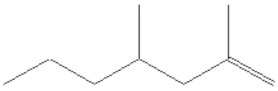
7.188	Cyclohexane,1,3,5-trimethyl-	C9H18	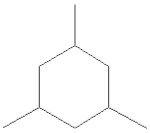
7.832	Cyclohexene,3,3,5-trimethyl-	C9H16	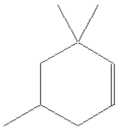
8.827	1-Octene,4-methyl-	C9H18	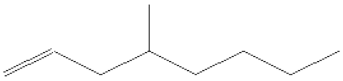

9.142	1,6-Octadiene,5,7-dimethyl-,(R)-	C10H18	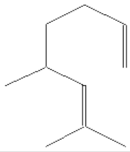
9.804	1,6-Octadiene,2,6-dimethyl-,(Z)-	C10H18	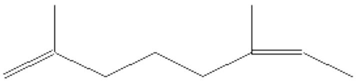
9.988	Cyclohexene,1-methyl-3-(1-methylethyl)-	C10H18	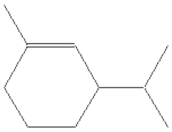
10.431	3,3-Dimethyl-6-methylenecyclohexene	C9H14	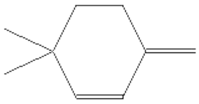
11.036	1-Undecene,10-methyl-	C12H24	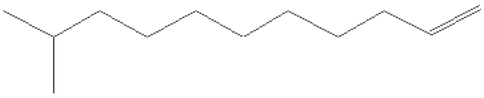
11.193	3-Heptene,4-propyl-	C10H20	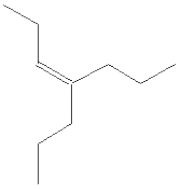
11.526	Decane,4-methylene-	C11H22	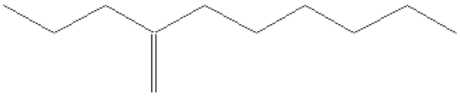
11.745	1-Nonyne	C9H16	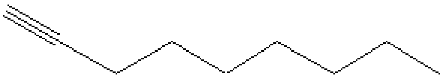
12.052	2-Octene,3,7-dimethyl-,(Z)-	C10H20	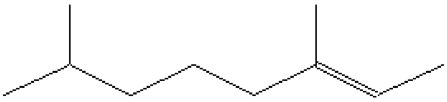
12.280	Benzene,1-ethyl-2-methyl-	C9H12	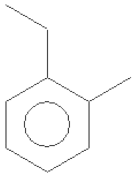
12.390	5-Undecene,8-mehtyl-,(E)-	C12H24	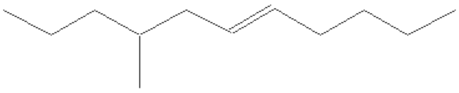
12.679	Benzene,1,2,3-trimethyl-	C9H12	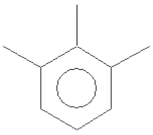
13.468	Cyclohexane,1-ethyl-4-(1-mehtylethyl)-,cis-	C10H18	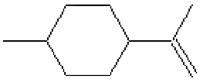

13.590	9-Methylbicyclo[3.3.1]nonane	C10H18	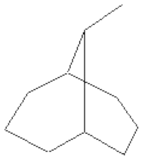
13.928	Nonane,2-methyl-3-methylene-	C11H22	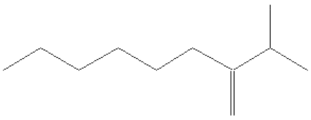
14.029	2-Decene,7-methyl-,(Z)-	C11H22	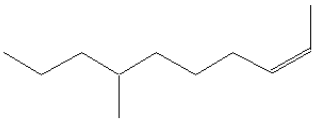
14.191	2-Decene,4-methyl-,(Z)-	C11H22	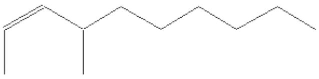
14.357	1-Nonene,4,6,8-trimethyl-	C12H24	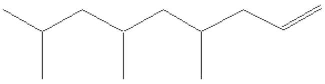
14.519	1-Decene,4-methyl-	C11H22	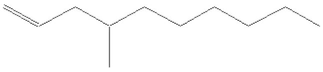
14.721	Nonane,2,6-dimethyl-	C11H24	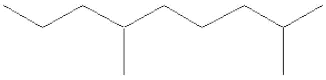
14.949	Decane,4-methyl-	C11H24	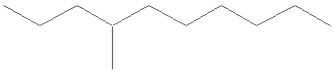
16.136	1-Undecene,7-methyl-	C12H24	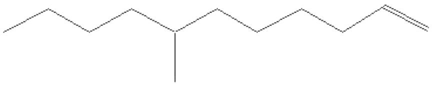
16.355	5-Tridecene,(Z)-	C13H26	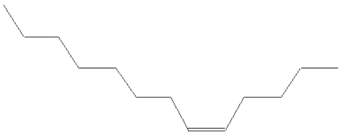
17.096	3-Tridecene	C13H24	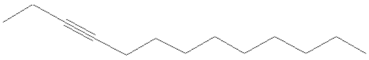
17.302	2-Decene,2,4-dimethyl-	C12H24	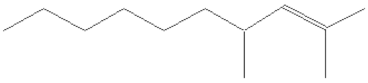
17.464	1-Decene,2,4-dimethyl-	C12H24	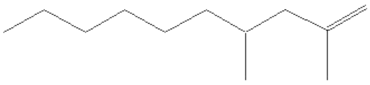
17.797	Hexane,1-(isopropylidenecyclopropyl)-	C12H22	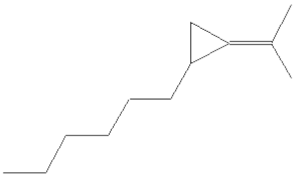

17.959	1,7-Nonadiene,4,8-dimethyl-	$C_{11}H_{20}$	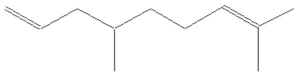
18.266	3-Tetradecene,(Z)-	$C_{14}H_{28}$	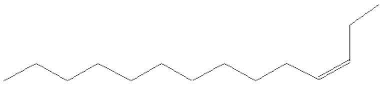
18.485	Cyclooctane,1,4-dimethyl,cis-	$C_{10}H_{20}$	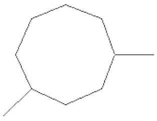
20.036	7-Octadecyne,2-methyl-	$C_{19}H_{36}$	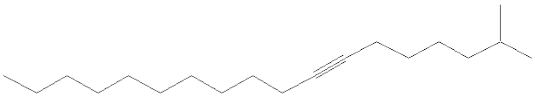
20.361	1,6-Octadiene,5,7-dimethyl,(R)-	$C_{10}H_{18}$	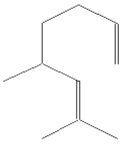
20.654	3-Hexadecene,(Z)-	$C_{16}H_{32}$	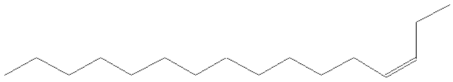
21.794	Cyclohexane,(2,2-dimethylcyclopentyl)-	$C_{13}H_{24}$	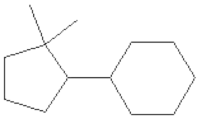
21.916	1,1’-Bicyclohexyl,2-propyl-,trans-	$C_{15}H_{28}$	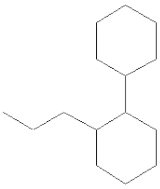
22.385	1-Isopropyl-1,4,5-trimethylcyclohexane	$C_{12}H_{24}$	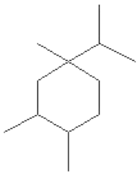
24.923	2-Undecene,4,5-dimethyl-[R*,R*-(E)]-	$C_{13}H_{26}$	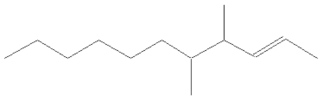
25.027	3-Octadecene,(E)-	$C_{18}H_{36}$	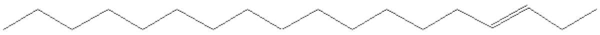
25.913	Decane,2,3,58-tetramethyl-	$C_{14}H_{30}$	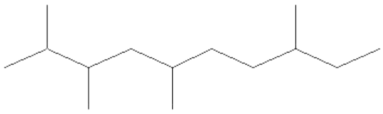
26.307	Dodecane,4,6-dimethyl-	$C_{14}H_{30}$	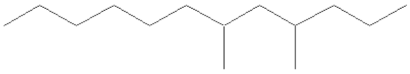
26.899	3-Eicosene,(E)-	$C_{20}H_{40}$	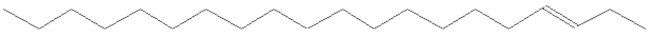
27.228	1-Docosene	$C_{22}H_{44}$	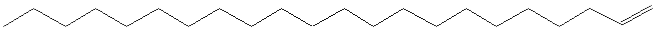
27.675	9-Eicosene,(E)-	$C_{20}H_{40}$	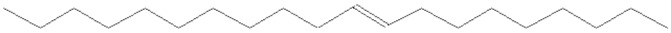

28.744	5-Eicosene,(E)-	C20H40	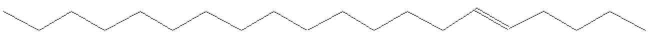
29.055	1-Octadecene	C18H36	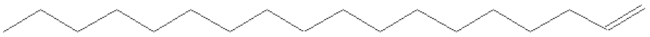
32.074	1,19-Eicosadiene	C20H38	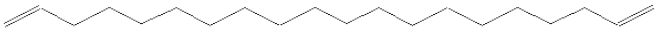
32.815	7-Octadecyne,2-methyl-	C19H36	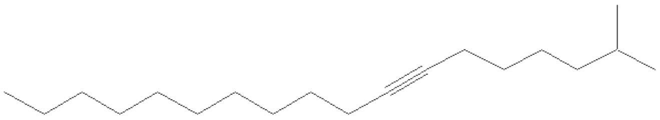
34.778	10-Heneicosene (c,t)	C21H42	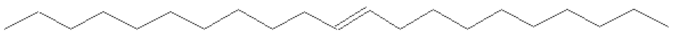
35.711	Eicosane	C20H42	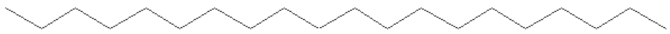
38.104	Cyclodocosane, ethyl-	C24H48	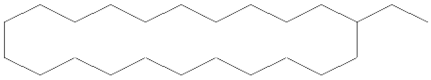
39.585	9-Tricosene,(Z)-	C23H46	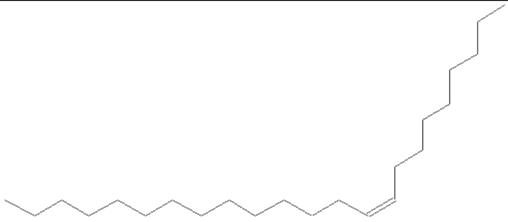
48.525	1,21-Docosadiene	C22H42	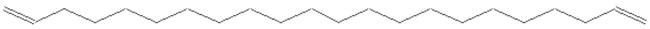
53.179	Cyclotetracosane	C24H48	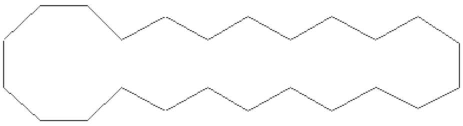
54.068	9-Hexacosene	C26H52	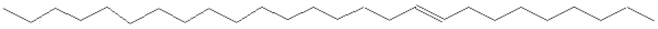
55.462	Cyclohexane,1,35 -trimethyl-2-octadecyl-	C27H54	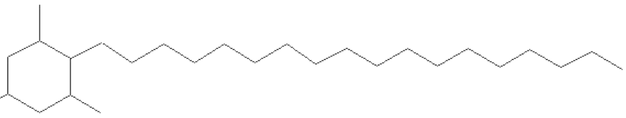
59.642	17-Pentatriacontene	C35H70	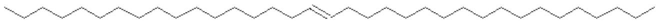

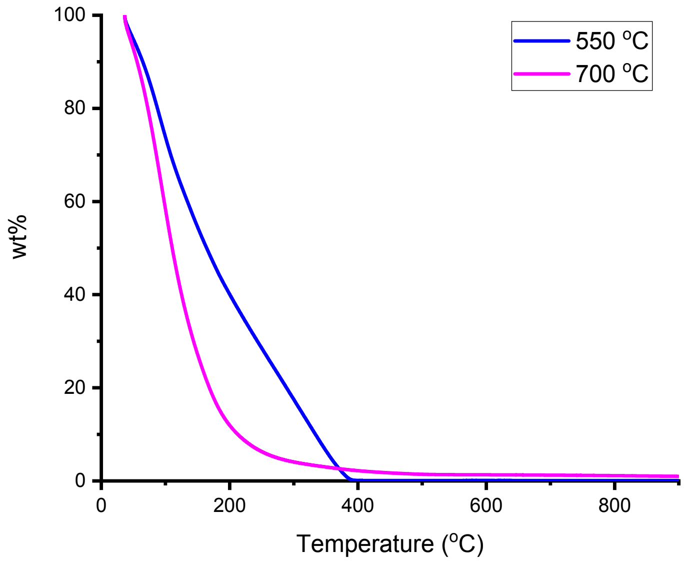

Figure S2. Effects of noncatalytic pyrolysis temperature on liquid distillation profile by TGA in N_2 (Feed: PP, 1st pyrolysis reactor: 100 g sand (45-100 μm), 2nd aromatization reactor: empty, N2 flow: 0.66 L/min, TOS = 2 h).

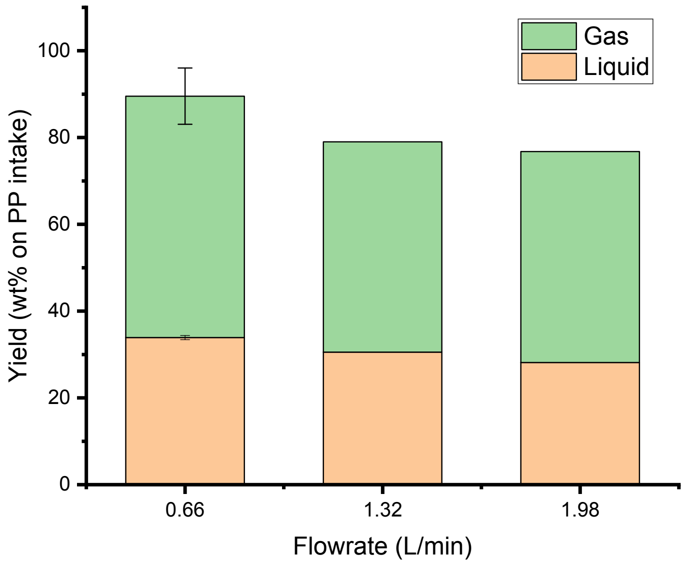

Figure S3. Effects of N_2 flowrate on gas and liquid yield (wt% on PP intake) (all reactor temperatures: 550 °C, 1st pyrolysis reactor: 100 g sand (45-100 μm), 2nd aromatization reactor: 100 g H-ZSM-5(38) catalyst (45-100 μm)).

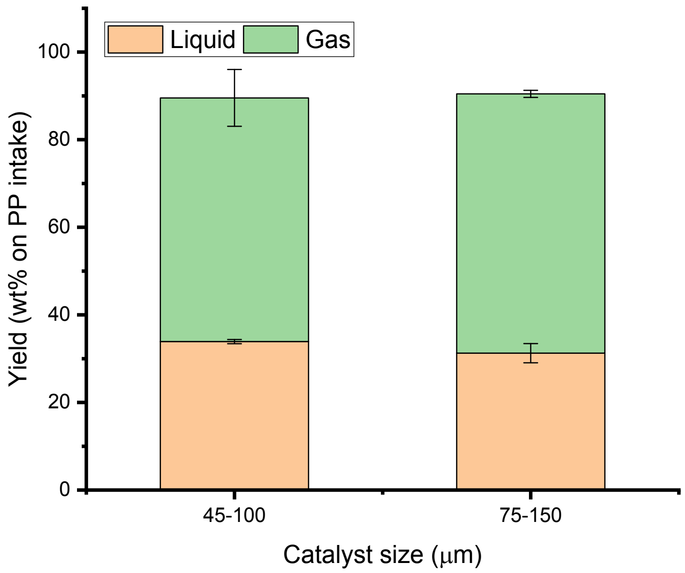

Figure S4. Effects of catalyst size on mass balance (conditions: all reactor temperatures: 550 ^{o}C, 1st pyrolysis reactor: 100 g sand (45-100 μm), 2nd aromatization reactor: 100 g H-ZSM-5(38) catalyst, N_2 flow: 0.66 L/min).

Section S2. Thiele modulus and effectiveness factor calculations

It is assumed the aromatization is a first-order reaction in a spherical catalyst pellet in the second aromatization reactor. 143 g PP or hexane (MW=84 g/mol) is converted into benzene (MW=78 g/mol) using 100 g H-ZSM-5 (38) catalyst of two different sizes: 45-100 and 75-150 μm. Time on stream is 1 h.

Reaction: C_6H_{12} = C_6H_6 + 3 H_2

For R_1=72.5 μm=72.5*10^(-6) m catalyst:

Measured Rate (obs) (mol/g-cat s) r'_{A1}=143*0.223/78/(60*60*100)=1.136*10^(-6) mol/g-cat s

For R_2=112.5 μm=112.5 * 10^(-6) m catalyst:

Measured Rate (obs) (mol/g-cat s) r'_{A2}= 143*0.189/78/(60*60*100)= 0.963*10^(-6) mol/g-cat s

Taking the ratio of the Thiele moduli for two runs [5]:

$$\frac{\phi_{11}}{\phi_{12}} = \frac{R_1}{R_2} = \frac{72.5}{112.5} \qquad \text{(Eq.S1)}$$

Combining with following equation:

$$\frac{-r'_{A2}R_2^2}{-r'_{A1}R_1^2} = \frac{\phi_{12}\coth\phi_{12}-1}{\phi_{11}\coth\phi_{11}-1} \qquad \text{(Eq.S2)}$$

The left of Eq.S2 is: -0.963*0.000001*112.5*112.5/(-1.136*0.000001*72.5*72.5)= 2.041

Substituting for ϕ_{11} in Eq.S2 by Eq.S1, the equation with one unknown is solved using Matlab.

vpasolve((x*coth(x) - 1)/(72.5/112.5*x*coth(72.5/112.5*x) - 1) == 2.041,x,[0 10],'random',true)

The Thiele modulus ϕ_{12} is calculated to be 2.613 and ϕ_{11} is 1.684.

The corresponding effectiveness factors are calculated using Eq.S3:

$$\eta_2 = \frac{3(\phi_{12}\coth\phi_{12}-1)}{\phi_{12}^2} \qquad \text{(Eq.S3)}$$

η_2= 3*(2.613*coth(2.613)-1)/(2.613*2.613) = 0.721

η_1= 3*(1.684*coth(1.684)-1)/(1.684*1.684) = 0.851

Table S3. Summary of Thiele modulus and effectiveness factors calculations.

Catalyst size (µm)	45-100	75-150
BTX yield (wt%)	22.3%	18.9%
Thiele moduli	1.684	2.613
Effectiveness factors	0.851	0.721

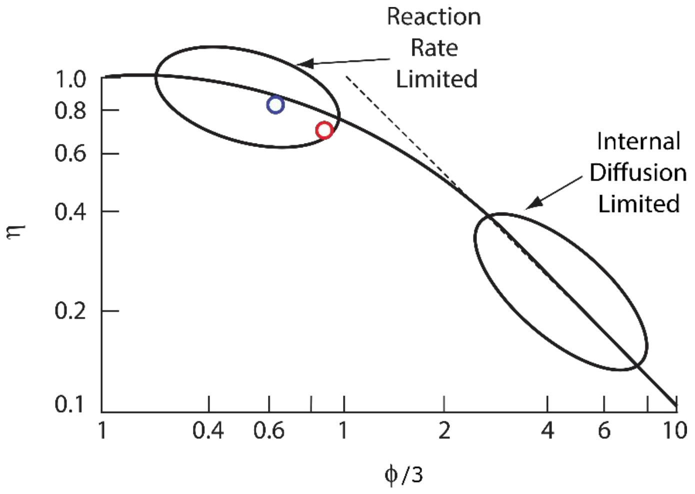

Figure S5. Relationship between Thiele modulus and effectiveness factor.:

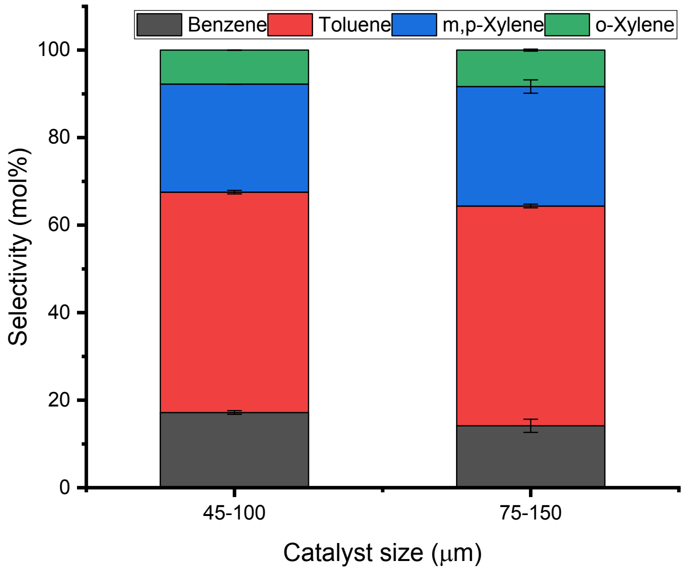

Figure S6. Effects of catalyst size on selectivity (conditions: all reactor temperatures: 550 °C, 1st pyrolysis reactor: 100 g sand (45-100 µm), 2nd aromatization reactor: 100 g H-ZSM-5(38) catalyst, N_2 flow: 0.66 L/min).

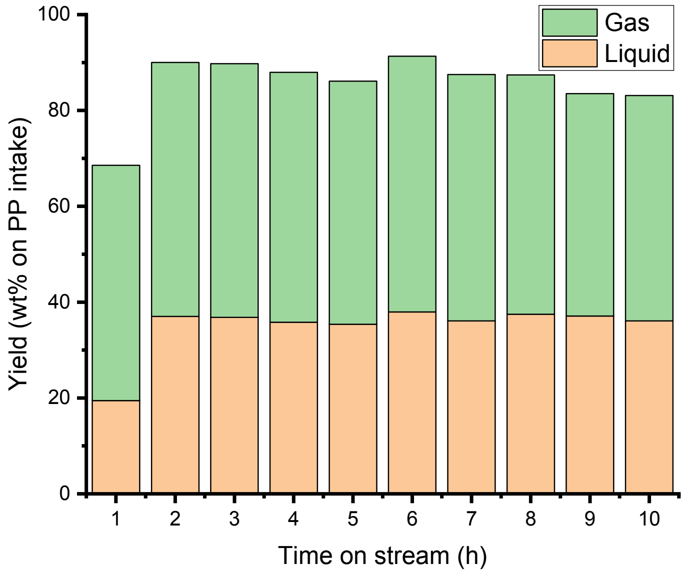

Figure S7. Gas and liquid yield (wt% on PP intake) as a function of TOS (all reactor temperatures: 550 °C, 1st pyrolysis reactor: 100 g sand (45-100 µm), 2nd aromatization reactor: 100 g catalyst (45-100 µm), N_2 flow: 0.66 L/min).

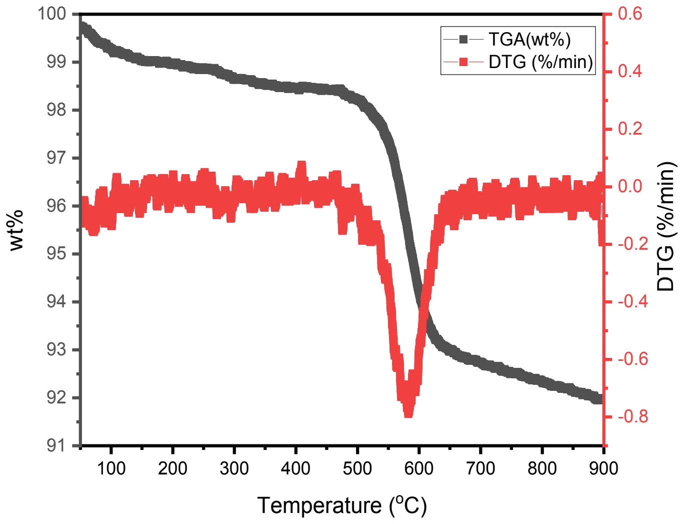

Figure S8. TGA and DTG curves of spent H-ZSM-5(38) catalyst after TOS = 10 h.

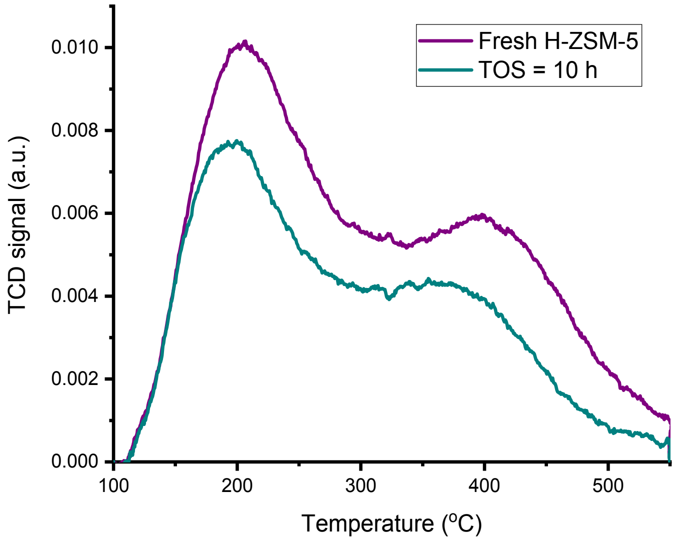

Figure S9. NH_3-TPD profiles of fresh and spent H-ZSM-5 catalyst after TOS of 10 h.

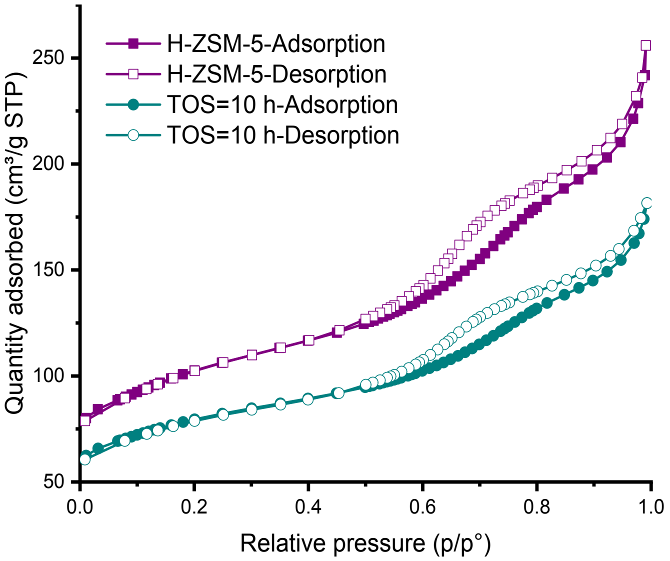

Figure S10. Nitrogen adsorption/desorption isotherms of fresh and spent H-ZSM-5 catalyst after TOS of 10 h.

References for supporting information

1. Grace, J.R., *Properties, Minimum Fluidization, and Geldart Groups*, in *Essentials of Fluidization Technology*. 2020. p. 11-31.
2. Bi, H.T. and J.R. Grace, *Flow regime diagrams for gas-solid fluidization and upward transport.* International Journal of Multiphase Flow, 1995. **21**(6): p. 1229-1236.
3. Bi, X., *Gas Fluidization Flow Regimes*, in *Essentials of Fluidization Technology*. 2020. p. 55-74.
4. Dekker, H.-J., *Catalytic conversion of glycerol to bio-based aromatics in a tandem fluidized bed reactor using an H-ZSM-5-based catalyst*. Master research project, University of Groningen, 2020.
5. Fogler, H.S., *Elements of chemical reaction engineering*. 2016: Prentice Hall.
